# An Effective Model-Based Voting Classifier for Diabetes Mellitus Classification

**DOI:** 10.3390/bioengineering13040480

**Published:** 2026-04-21

**Authors:** Diyar Qader Zeebaree, Merdin Shamal Salih, Danial William Odeesho, Dilovan Asaad Zebari, Nechirvan Asaad Zebari, Omar I. Dallal Bashi, Reving Masoud Abdulhakeem, Yahya Ahmed Yahya

**Affiliations:** 1 Department of Cyber Security Techniques Engineering, College of Computer and AI, Northern Technical University, Mosul 41001, Iraq; dqszeebaree@ntu.edu.iq (D.Q.Z.);; 2 Department of Computer Science, Cihan University-Duhok, Duhok 42001, Iraq; 3 Department of Computer Science, College of Science, Nawroz University, Duhok 42001, Iraq; 4 Department of Information Technology, Technical College of Informatics, Akre University for Applied Sciences, Akre 42002, Iraq; 5 Faculty of Computing and Information Technology, Sohar University, Sohar 311, Oman; 6 Department of Information Technology, Lebanese French University, Erbil 44001, Iraq; nechirvan.asaad@lfu.edu.krd; 7 Technical Engineering College for Computer and AI–Mosul, Northern Technical University, Mosul 41001, Iraq; 8 Computer and Communication Engineering Department, Nawroz University, Duhok 42001, Iraq; 9 Prosthetics & Orthotics Engineering, Al-Kitab University, Kirkuk 36001, Iraq

**Keywords:** diabetic prediction, PIAM and Frankfurt, feature selection, classification

## Abstract

Diabetes mellitus is a health issue that is rapidly increasing worldwide, and it affects more than 347 million people globally. It is important to note that the disease can be successfully detected in its early stages, enabling physicians to avoid complications and improve patient outcomes. Despite the fact that machine learning (ML) has been extensively used in diabetes classification, the available solutions tend to place little or no emphasis on feature selection and ensembles, which limits prediction accuracy and generalizability. In this study, we introduce a hybrid framework that is based on three feature-selection algorithms, specifically, genetic algorithm (GA), correlation-based feature selection (CFS) and recursive feature elimination (RFE), in single and hybrid forms, and three classifiers, namely, multi-layer perceptron (MLP), support vector machine (SVM) and random forest (RF), to achieve a greater predictive robustness with the aid of soft voting. Experimental findings obtained from a benchmark diabetes dataset indicate that the RFE + CFS + SVM combination achieves the best performance, with an accuracy of 98.0%, sensitivity of 97.43%, specificity of 99.03%, precision of 99.51% and F1-score of 98.72%. These results indicate that the suggested hybrid feature-selection and ensemble learning model can offer a robust and highly effective approach for early-stage diabetes diagnosis, one which clinicians may use to make timely and accurate decisions.

## 1. Introduction

Rapid urbanization and modernization have been responsible for the onset of a wide variety of chronic diseases, a development which poses a significant challenge to the world’s public health [1]. Since the beginning of modern medicine, it has been common knowledge that an accurate diagnosis is one of the most important factors in providing an effective treatment. The condition is often still diagnosed via a battery of physical and chemical examinations. The findings of the many diagnostic tests and examinations point to the presence of a certain ailment. There is a possibility of mistakes in this prediction because of the high uncertainty within the various parameters utilized for testing. When a disease is not diagnosed properly, there may then be no chance of finding a cure or effective management. Diabetes can be regarded as one of the most significant difficulties facing the medical profession on a global scale, and the disease’s impact is escalating at an extremely rapid rate [2]. This term refers to a collection of metabolic conditions that are characterized by elevated levels of sugar in the blood. Diabetes may put a person at risk of developing a number of other significant conditions over the course of their lifetime, including cardiovascular disease, stroke, kidney failure, heart attack, peripheral artery disease and disorders affecting the blood vessels and nerves [3]. According to recent research and statistics, there are currently 463 million individuals in the world who are afflicted with diabetes. This number is projected to rise to 578 million by the year 2030 and 700 million by the year 2045. Because of this, it is anticipated that the number of diabetic patients will increase by approximately 25% in the year 2030 and 51% in the year 2045 [1]. There are four basic types of diabetes known to exist. These include type 1, type 2, gestational diabetes, and additional variants. Type 1 and type 2 diabetes are the most frequent forms of diabetes. Type 1 diabetes is more common in younger people, particularly children and adolescents. In this particular circumstance, the body generates either extremely small amounts of insulin or none at all. As a direct consequence of this, daily insulin injections are required in order to maintain adequate glucose control. This form of diabetes is characterized by a wide range of symptoms, including increased urination frequency, rapid weight loss, abnormal thirst, unrelenting hunger, blurred vision, and fatigue. Insulin therapy is one method that can be used to treat this condition. The majority of people who develop type 2 diabetes are adults, accounting for approximately 90% of cases. Higher blood-glucose levels are the result of the body’s inability to properly respond to insulin. People who are overweight, have poor diets, have high blood pressure, or do not engage in sufficient physical activity are regarded as being associated with important risk factors for developing type 2 diabetes. When oral medication is not sufficient to manage blood-glucose levels, a diabetic may need to take insulin via injection [4,5].

The number of diabetes patients has been steadily growing over the past few years, which has been a challenge for the medical sector. Recent developments in the field of healthcare have led to improvements in the early diagnosis of diabetes. However, nearly half of the people with diabetes are unaware that they have the condition. The diagnosis may not come for more than ten years in some cases. In the event that treatment is delayed, serious health issues such as kidney failure, an increased risk of blindness, high blood pressure, nerve damage, and stroke may emerge. At present, diabetes is a disease that cannot be cured, and the effectiveness of treatment for diabetes is mostly dependent accurate diagnosis and prompt treatment. When diabetes is detected in its early stages, it is possible to gain control over the condition. On the other hand, diabetes can cause substantial damage to the body and make the condition difficult to treat if it is undiscovered or left untreated. However, early diabetes detection can lead to improved treatment, resulting in decreased morbidity and mortality [6].

The majority of medical data is nonlinear, non-normal, correlation-structured, and complicated in form [3], which makes the analysis of diabetic data a challenging task. The early diagnosis and prediction of the disease are both currently done manually by a doctor based on their expertise, experience, and observations. At this time, there is no fully automated method that has been widely adopted. Although the healthcare sector already collects a vast amount of data, this does not guarantee that inherent latent patterns will be revealed. As a consequence of this, the decisions that are made manually can be extremely deceptive and hazardous, particularly when it comes to early diagnosis. This is because some parameters may go unnoticed, which can have a disastrous effect on the observations and outcomes [1]. Researchers, over the course of the past several years, have utilized a wide variety of technologies and algorithms in an effort to establish a method for diagnosing diabetes. One of these technologies is known as machine learning (ML), and another is known as deep learning (DL). Due to the rapid rise in the number of cases of diabetes and the increased complexity of the enormous data records of diabetic patients [7], it is becoming increasingly difficult for medical professionals to provide efficient treatment using manual approaches. Because of this, medical data mining can be successfully used for the purpose of improving the diagnosis of a diabetic patient. This is because it permits the detection of diabetes at an earlier stage. The data categorization method can be utilized to differentiate between patients diagnosed with diabetes and those who do not have the condition.

Despite this, raw unstructured datasets can contain a number of attributes that are both useless and unclear. The overall effectiveness of classification in data mining is hindered by the presence of such attributes, which contributes to the problem. Therefore, an efficient attribute optimization approach can be utilized, eliminating less-important features and producing an optimized dataset with important symptoms that can be mined accurately using a suitable classification algorithm. This dataset will contain the key symptoms. The attribute optimization method is an optimizing agent that can be successfully applied to huge and complicated datasets to lower the sample size without sacrificing any vital data. This is possible because the method for optimizing attributes functions as an optimizing agent. As a result, attribute optimization reduces the amount of time required for the classification process while simultaneously increasing its accuracy.

Even given the extensive amounts of research on individual classifiers and feature-selection algorithms, the originality of the proposed research is in the simultaneous consideration of various feature-selection methods, combined with ensemble learning, which is used to provide a systematic framework for diabetes classification. This integration facilitates enhanced predictive performance, reduces feature redundancy, and guarantees better generalizability across datasets, a factor which has not been adequately investigated in previous studies

The fundamental purpose of this research is to diagnose diabetes in its initial stages so that patients can obtain prompt treatment and avert the serious consequences that are associated with this life-threatening condition. In addition, another purpose of this research is to achieve a high level of classification accuracy. This study contributes significantly to the field of research pertaining to the diagnosis of diabetes. The solution arrived at has achieved a high degree of classification accuracy when applied to diabetes prediction. This study has demonstrated higher level of accuracy in classification when compared to other investigations. The following is a high-level summary of the primary contributions that this work makes:

• This research develops an enhanced model of predicting diabetes at an early stage by incorporating advanced preprocessing techniques.
• This work presents hybrid methods of feature selection (GA + CFS, GA + RFE and RFE + CFS) to select the most informative and relevant features.
• A new type of soft voting ensemble classifier (MSR) is proposed, which is an integration of multiple base classifiers that improves predictive stability and accuracy.
• Two benchmark datasets (PIAM and Frankfurt) and their combined dataset are used in the experiments to evaluate the generalization and reliability of the model.

## 2. Related Works

The classification of diabetes types is among the most difficult problems that medical professionals face, and it requires the completion of several different tests. However, diagnosing diabetes based on the analysis of several factors at one time may lead to erroneous results. Thus, diabetes classification is considered a challenging task. Recent technological advancements, particularly those in the field of machine learning, have shown themselves to be extremely advantageous for the healthcare sector. Numerous research-based diabetes classification models have been proposed in the literature.

An artificial neural network (ANN) model was proposed by Pradhan et al. [8] in 2020 as a method for diagnosing diabetes in individual patients. The performance of the suggested model was evaluated with the help of the Pima Indians with Diabetes (PID) dataset. In the preprocessing stage, data normalization was performed. After that, the ANN was trained using the data from the training session. In the end, the performance of the model was assessed with the use of testing data. The accuracy of the model increased to 85.09% when it was trained with 70% of the data and tested with 30% of the data. Gupta et al. [9] used the naive Bayes and support vector machine classification methods in 2021 for the purpose of diagnosing diabetes. They used the Pima Indians with Diabetes dataset. In addition to this, they improved the accuracy of the model by the use of a feature-selection-based technique and k-fold cross-validation. The results of the experiments demonstrated that the naive Bayes model performed worse than the support vector machine. On the other hand, neither the attained accuracy nor the comparison to the state of the art are included in this comparison. Barik et al. [10] referred to the Pima Indians with Diabetes dataset in their work from 2021. The authors trained the model by utilizing eight of the total nine attributes that are provided in the dataset, using Jupyter Notebook as an integrated development environment (IDE). RF and XGBoost were the algorithms that were implemented. Then, the models were trained once the hyperparameters were initially specified. The RF classifier was able to achieve an accuracy of 71.9% after being improved. The hybrid model that was suggested using XG boost achieved a level of accuracy of 74.1 percent. When compared to the models that had previously been made available, the accuracy attained by the models in question was noticeably inferior. In order to get the most out of the algorithm, the tuning of the hyperparameters needs to be improved. A system for the prediction of diabetes based on soft computing was presented by Kumari et al. [11] in 2021. This system makes use of three supervised machine learning techniques that are utilized in an ensemble fashion. In order to conduct the evaluation, they made use of the Pima Indians with Diabetes dataset and a breast cancer dataset. They used random forest, logistic regression, and naive Bayes, and compared their system’s performance to those of state-of-the-art individual and ensemble techniques. Their system surpassed the competition, achieving an accuracy rate of 79%. Using the PIDD dataset, Abdulhadi et al. [12] developed a number of machine learning models in 2021 to predict the existence of diabetes in women. The models described above were used to make the predictions. The authors solved the problem of missing data by employing a method known as mean substitution, and they rescaled all of the characteristics by employing a technique known as standardization. The models were constructed using LR, linear discriminant analysis (LDA), support vector machines (both linear and polynomial), and RF. According to the findings presented in the research, the RF model was able to obtain a level of accuracy that was as high as 82%.

Khanam et al. [13] conducted research in 2021 evaluating several approaches to machine learning, with the goal of diagnosing diabetes in its early stages. A diabetes dataset was utilized by the researchers so that they could evaluate the effectiveness of various algorithms. Seven different machine learning methods, including support vector machine (SVM), random forest (RF), logistic regression (LR), k-nearest neighbors (kNN), and neural network (NN), were utilized. On the Pima Indians with Diabetes (PID) dataset, linear regression (LR) and support vector machine (SVM) both performed well, but a neural network (NN) surpassed the other techniques and achieved an accuracy of 88.6%. An end-to-end healthcare monitoring platform was presented by Ramesh et al. [14] in the year 2021. The framework was designed to manage diabetes and anticipate high-risk cases using the Pima Indians with Diabetes dataset. They achieved an accuracy of 83.20 percent, a sensitivity of 87.20 percent, and a specificity of 79 percent, respectively. In 2021, Azad et al. [15] introduced a model PMSGD to classify diabetes. In the model that was proposed, the synthetic minority oversampling technique (SMOTE), the genetic algorithm (GA), and DT were utilized. The model that was proposed was built using a total of four layers. The initial layer consisted of the performance of data preparation operations. In the second layer, the features that worked best for training were selected. In the third layer of training, the model was developed. In the fourth layer of the evaluation, the performance of the model was analyzed using a variety of performance matrices. The accuracy of the model was measured using the Pima Indians with Diabetes (PID) dataset, and the results showed that it had an accuracy of 82.1256%.

Past studies have also expanded into machine learning and AI prediction used for the risk assessment of diabetes. As an illustration, Lugner et al. (2024) [16] used UK Biobank data to apply the ML techniques to find the best predictors of type 2 diabetes, which demonstrates the potential of big-data-driven solutions in disease screening. Trends and gaps in the application of MLs to predict T2DM have also been named in systematic studies in the last three decades, such as changes towards ensemble and deep learning models, as described in Zhao et al. (2025) [17]. Optimized model selection and class imbalance management have been suggested as parts of frameworks used to enhance predictive robustness, as outlined in Kiran et al. (2025) [18], and systematic reviews point to the increased applicability of ML in prognosticating the transition of gestational diabetes to type 2 diabetes, as described in Abousaber et al. (2025) [19]. Moreover, the studies on genetic biomarkers analyzed by means of ML techniques highlight the growing breadth of the feature-based approaches used for prediction in the study of diabetes, as described in Khan et al. (2024) [20]. Some of the recent precedential works used in diabetic classification have been summarized in Table 1.

The recent works in the literature show serious issues relating to the validation of machine learning models in clinical practices. A systematic review by Tan et al. (2023) [33] found that most of the current models for diabetes complications are only internally validated and have a high probability of bias, which limits their application in clinical real-world practice. In the same vein, a recent meta-analysis by Li et al. (2025) [34] has shown that despite the high predictive performance of machine learning models, few studies use external validation, and as a result, some studies tend to overestimate the effectiveness of models. To overcome these shortcomings, in recent studies more emphasis has been placed on sound validation strategies. Importantly, a machine learning model using real-world clinical data was developed and externally validated by Du et al. (2025) [35], which emphasizes the significance of testing model generalization in applications involving independent datasets. Moreover, Liu et al. (2025) [36] highlighted the importance of prospective validation as the means of guaranteeing the stability and reliability of the model in the long run. Further, recent studies have also sought better model interpretability and clinical usability. The application of transparency in clinical decision-making was shown to be important by Tuinte et al. (2025) [37], who suggested an interpretable machine learning framework verified across multiple centers. Similarly, He et al. (2024) [38] applied massive clinical and metabolomic data with external validation, which supports the necessity of strong assessment in different populations. Although many of the early works claim encouraging accuracy results with data sources like the Pima Indians with Diabetes dataset, such models frequently make use only of train–test splits or internal validation. This drawback is material, because among recent systematic reviews, Tan et al. (2023) [33] note that this type of approach carries a high risk of bias and overfitting, decreasing clinical reliability.

Although the development of machine learning in the prediction of diabetes has achieved significant progress, there are major limitations. The current research is mostly based on one dataset (Pima Indians with Diabetes) and applies internal validation methods, which can lead to overestimation of model performance. Moreover, recent findings indicate that the lack of external validation and insufficient analysis of training dynamics may lead to model findings that do not generalize well to real-world clinical settings. Also, most of the studies are concerned with accuracy, and are not concerned with other clinically relevant measures, including sensitivity and the balance between degrees of specificity. These shortcomings highlight the need for a robust and highly validated framework to ensure generalization, reduce overfitting, and offer accurate clinical decision support. This study contrasts with previous research, which tends to use only one feature-selection algorithm or test the effectiveness on a single dataset, by focusing on hybrid feature selection, robust ensemble-based decision-making, and multi-dataset validation. Accuracy is the major reporting characteristic associated with most of the current methods, but in clinical screening applications, balanced sensitivity and specificity are vital characteristics of the study, as reported in this paper.

## 3. Proposed Framework

**Figure 1:**
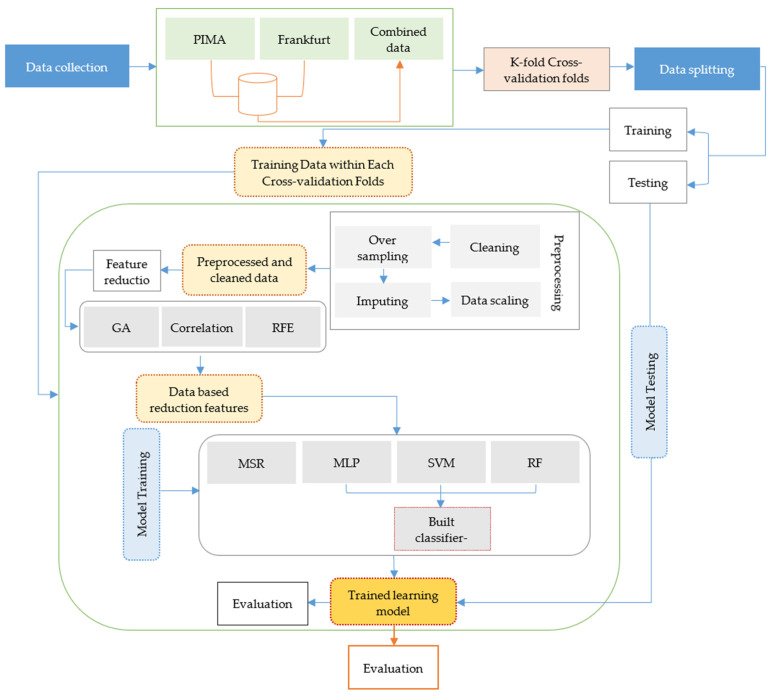
Overview of proposed framework.

The proposed framework will offer a reliable and interpretable framework for diagnosing diabetes at the initial stage. As shown in Figure 1, the framework is composed of four major steps, namely, preprocessing the data, feature selection, classification, and performance evaluation. Preprocessing helps in ensuring the quality and consistency of the input data, which is essential for effective model training. To improve the extraction of relevant features and to eliminate redundancy, three established algorithms, genetic algorithm (GA), correlation-based feature selection (CFS), and recursive feature elimination (RFE), are used alone and in varying combinations (GA + CFS, GA + RFE, and RFE + CFS).

Three traditional machine learning models are used to classify; these are multi-layer perceptron (MLP), support vector machine (SVM), and random forest (RF). The new ensemble classifier (MSR) integrates multiple base classifiers by soft voting in order to achieve improved predictive robustness. This results in a comprehensive framework with which to evaluate the performance of diabetes classification in a variety of model–feature combinations. By combining feature selection and ensemble learning in a unified pipeline, the framework provides a systematic, interpretable, and generalizable approach, addressing limitations of previous studies that focused on single classifiers or individual feature-selection methods.

The primary innovation of this work is the integrated approach of hybrid feature selection and ensemble classification. Through a systematic assessment of the various FS combinations, and using classifiers including classical classifiers and a new MSR ensemble, the framework provides insights into the most effective combinations of feature sets and models for reliable diabetes prediction.

Data splitting and cross-validation are carried out before any preprocessing steps in order to avoid data leakage. The imputation of all missing values, oversampling, standardization and feature selection is all done on the training folds only. Validation folds are maintained independently at all times and do not ever undergo preprocessing or training. This ensures a strict separation between training and validation data, and will ensure fair performance assessment.

### 3.1. Dataset

Two different datasets are utilized in the training and testing of the proposed diabetic classification model. The following sections will provide details regarding the dataset that was utilized.

#### 3.1.1. The Pima Indians with Diabetes Dataset

The Pima Indians with Diabetes dataset (PID) served as one of the primary research data sources. The primary reason for utilizing the Pima Indians with Diabetes dataset is that a majority of the world’s population now adheres to a lifestyle that is characterized by an increased reliance on processed foods and a decrease in the amount of time spent engaging in physical activity. Because of the high probability of developing diabetes, the National Institute of Diabetes and Digestive and Kidney Diseases (NIDDK) has been conducting the PID long-term cohort study since 1965. The dataset includes several diagnostic parameters and measurements that, when applied to the patient, make it possible to determine in advance whether the individual is suffering from diabetes or another type of chronic disease. PID only accepts participants who are at least 21 years old, and all participants are female. PID consists of a total of 768 instances, of which 268 samples were found to be diabetic and 500 samples were found to be non-diabetic [2, 6]. The patient’s history of diabetes, their body mass index (BMI), their insulin level, their age, their blood pressure, the thickness of their skin, and their glucose levels were the factors that had the most impact on the accuracy of the prediction of diabetes. The Pedigree Function is included as an output feature (Table 2).

This study made use of a diabetic dataset obtained from (https://www.kaggle.com/uciml/pima-indians-diabetes-database, accessed on 15 February 2025). The high prevalence of type 2 diabetes in the Native American Pima tribe living in the region that is now known as central and southern Arizona is the reason why the Pima Indian dataset was selected. Because of their genetic predispositions, this tribe has been able to sustain a traditional way of life on a diet that is low in carbohydrates. Because of the rapid transition from traditional crops to manufactured foods, the Pima tribe has seen an increase in the prevalence of diabetes in recent years.

#### 3.1.2. Frankfurt

There are 2000 records in the Frankfurt Hospital (Germany) dataset [39], and each record has nine properties (https://www.kaggle.com/johndasilva/diabetes?select=diabetes.csv, accessed on 15 February 2025). Table 2 provides a concise summary of these characteristics, with the ninth item serving as an indicator of whether or not the disease is present. This item is considered the target variable, namely, the disease’s absence or presence (value of 0 or 1, respectively). Given the facts that all of the patients are females and their ages range from 21 to 81, 32.4% of the records have a value of 1, while the remaining 67.6% of the records contain a value of 0. The first attribute, Pregnancies, represents the number of pregnancies that have occurred, with a range that goes from 0 to 17. The Glucose attribute is the result of the glucose tolerance test, which measures how the body transports sugar from the blood into tissues such as muscle and fat; values range from 0 to 199. The measurements of blood pressure, also known as arterial pressure, are the pressures that are felt in the arteries when the heart is at rest in-between beats; the values that have been reported for blood pressure have ranged from 0 to 122. Insulin is a hormone that helps transport glucose, often known as blood sugar, from the bloodstream into the cells of the body. The readings for Insulin range anywhere from 0 to 864. The Skin-Thickness attribute gives information about the body’s fat reserves; its possible values range from 0 to 99. The Body Mass Index (BMI) factor provides a method that is both fast and reliable when used to determine if a patient is obese or underweight. It was reported with values ranging from 0 to 67.1 at various points in time. The Diabetes Pedigree Function, which offers a summary of the history of diabetes mellitus in relatives as well as the genetic relationship of those ancestors to the subject, can take float values anywhere between 0.078 and 2.42.

### 3.2. Data Preprocessing

Data preprocessing is accomplished through the execution of a series of sub-operations, each of which comprises many processes that can be performed on datasets in preparation for analysis and modeling. Preparation of the data has always been regarded as the single most important stage in any data-driven analysis. Preprocessing deals with understanding the data, developing a better understanding through the visualization of different aspects of it, and preprocessing data also takes care of problems such as redundant and imbalanced data, highly correlated and low variance attributes in the dataset, missing values, and outliers in the dataset [40]. The dataset that was utilized is referred to as “messy,” which means that it contains both missing and erroneous data. For this reason, the primary focus was placed on data wrangling and cleaning. Following the completion of the preprocessing step, it was discovered that the dataset includes a significant number of missing values, as well as outliers, and that the values of the attributes are not normalized. In this work, the approach used for preprocessing the data consisted of data cleaning, data imputation, data balancing, and the normalization of the data. Inconsistencies and noise, also known as the determinations of improper data, were removed throughout the process of cleaning the data. For the purpose of data imputation, the missForest method was utilized. After that, the SMOTE and ADASYN techniques were employed to solve the problem of imbalanced data by generating synthetic samples from minority group samples and their neighbors’ samples. This allowed the problem to be resolved. As a result, the information normalization methodology was applied to the process of converting all of the variable values into a specific range in order to obtain robust links between them.

#### 3.2.1. Data Cleaning

Data cleaning is the first step in the preprocessing stage, and it involves eliminating records and attributes that are not necessary for the present analysis. The dataset contains a number of categorical attributes that require removal for reasons related to privacy and data relevance [41]. In addition, the dataset lacks values for some patients’ diabetes types, which is important information for this study because we investigated diabetes complications in diabetic patients. These missing values pose a problem because the dataset is relied upon for this important information. As a result, all instances that were affected by this issue were eliminated.

#### 3.2.2. Missing Data Value Imputing

When training classifiers, dealing with missing values is critical because the majority of the existing machine learning techniques cannot be used with data that contain missing values. Only the nationality variable in our dataset contains categorical values, and it is this attribute that caused these kinds of problems. The dataset contained missing values that were missing at random. When data are missing at random, it signifies that the propensity for an instance to be missing is tied to the data that is present in the dataset rather than the data that is missing. Therefore, it is possible to predict the missing value for a variable based on the values of the other variables. For instance, HbA1c values that are missing may be lower or greater than what they would have been if they had been truly measured. This is due to the fact that HbA1c depends on age, duration of T2DM, fasting plasma glucose, and a few other values as well. The existence of missing values in a dataset results in the elimination of a sizeable quantity of the data’s associated information. To have a deeper understanding of the data at hand, it is necessary to impute the values of any missing data using the appropriate computational procedures. Some straightforward approaches to imputations include the replacement of values with the value zero, the elimination of complete instances, the utilization of mean imputation and KNN [41,42].

Mean imputation is a statistical method that, in its most basic form, replaces any missing value in an attribute with the average of the observational data for that characteristic in other records or cases. Mean imputation has a number of potential downsides, one of which is that it can produce biased findings. In addition, KNN imputation has a few drawbacks, including that it is vulnerable to outliers due to its utilization of the Euclidean distance below the surface, and it is unable to be implemented for categorical variables since it requires a few forms of transformation to numeric values. Therefore, employing these procedures is not appropriate in this context, as these techniques are considered inefficient for this dataset

MissForest is an imputation technique based on machine learning. In order to complete the task, it makes use of a random forest algorithm. Because it follows an iterative approach, each time through the process, the predictions that are generated become more accurate. Iteratively applying random forest models, this approach fills in missing data using imputation techniques. The initial step of this technique is to choose the attribute that has the fewest missing values. After the selection has been made, the mean of the candidate column is used to fill in any missing values in the candidate column. Additionally, the candidate column is then fitted to a random forest technique and considered as the output, while other columns in the dataset are considered as inputs to the technique. This is done in contrast to the first step, in which the candidate column was treated as the output. Imputing missing rows for the candidate column, utilizing prediction from the fitted random forest, is done after the random forest technique has been trained. Continuing in this manner will ensure that all other columns in the dataset have been covered. The fact that this technique does not need considerable data processing is the main advantage of using it. A random forest technique can identify which features are significant. Therefore, it can identify which features are most significant. In addition, it does not need any adjusting, unlike the k-nearest neighbors algorithm. Last but not least, random forest is capable of dealing with categorical data types, despite the fact that it does not care about them. A description of the missForest approach can be obtained by referring to pseudo-Algorithm 1 [43].
**Algorithm 1:** Impute missing values.**Require:** A with a matrix of *a* × *b*, stopping criterion *γ***Step 1.** Make an educated approximation about the values that are missing.**Step 2.** ***z*** ← *a* vector containing the sorted indices of each column in *A*, *w.r.t.* increasing number of values that are missing;**Step 3.** while not γ do**Step 4.** *A^i^_old_* ← storing a matrix that was originally imputed;**Step 5.** for *k* in *z* do _(_*_k_*_) (_*_k_*_)_**Step 6.** Construct a random forest: *n_obs_* ∼ *m_obs_*;**Step 7.** Predict *n*^(^*^k^*^)^ using *m*^(^*^k^*^)^;**Step 8.** *A^i^* ← update imputed matrix, using predicted *n*^(^*^k^*^)^;**Step 9.** end for**Step 10.** update *γ*.**Step 11.** end while**Step 12.** return the imputed matrix *A^i^*

The variation between the newly imputed data matrix and the former one increases for the first time with regard to both variable kinds; if both types of variables are available, the stopping criterion *γ* is satisfied. The difference between the two sets of continuous variables, *Z*, is defined as follows:

(1) $$\nabla Z=\;\frac{\sum_{j\in Z}{\left(A_{new}^{i}-A_{old}^{i}\right)}^{2}\;}{\sum_{j\in Z}{\left(A_{new}^{i}\right)}^{2}\;}$$

and for the set of categorical variables *Y* it is defined as

(2) $$\nabla Y=\frac{\sum_{j\in Y}\sum_{i=1}^{z}I_{A_{new}^{i}\neq A_{old}^{i}}}{\;\#ZA}$$

where #ZA is the number of missing values in the categorical variables.

#### 3.2.3. Imbalanced Data Oversampling

The data is considered to be imbalanced when the classes do not have a proportional distribution. A majority of real-world datasets exhibit an imbalance in their data, in which normal data has a significantly higher frequency of occurrence in comparison with abnormal samples. In general, classifiers applied to these datasets may suffer from overfitting or underfitting. In addition to random sampling with replacement, there are two common methods that are used to oversample minority classes, including the Synthetic Minority Oversampling Technique (SMOTE) and the Adaptive Synthetic (ADASYN) sampling method [44].

The SMOTE method is an oversampling method that involves the generation of synthetic samples for the underrepresented group. The overfitting problem caused by random oversampling can be solved with the help of this approach. It focuses on the feature space in order to produce new examples with the assistance of interpolations between the positive instances that lie together in close proximity. First, N, the total number of synthetic observations generated through oversampling, is specified. In most cases, it is chosen in such a way that the binary class distribution is 1:1. However, this could be adjusted according to the requirements. The next step in the process is to initiate the repetition by randomly selecting a minority class instance. After that, the KNNs for that instance are retrieved; the default value is 5. Finally, N samples are selected from these K neighbors to serve as interpolation points for generating new synthetic instances. To accomplish this, the difference in feature space between the selected instance and its neighbors is computed using an appropriate distance metric. Then, this difference is multiplied by any random number between 0 and 1, and the result is added to the first feature vector. This oversampling procedure is performed using Algorithm 2, the SMOTE algorithm.
**Algorithm 2:** SMOTE**Require:** Select values for *x* and *y*, where *x* and *y*, respectively, stand for the number of synthetic observations and the number of neighbors with the closest distance.**Step 1.** Let us designate by *Z_a_* the observations that belong to the minority class, with *B* = 1 through *m_n_*, and let us denote by *R* the set of all *Z_a_*, such that R ∋ Z*_a_*. For every Z*_a_*.**Step 2.** In order to determine *Z_a_* of k-nearest neighbors, it must first compute the Euclidean distance that separates it from every other element in *R*.**Step 3.** Let *N_ax_* represent the set of the k-nearest neighbors of *Z_a_*.**Step 4.** Take a randomized sample of *Y* synthetic observations, abbreviated *Z_ab_*, from *N_ax_* with replacement. The *b* value ranges from 1 to *Y*.**Step 5.** Let us use the symbol *λ* to signify a number between 0 and 1. To make a synthetic observation using the formula *Z_x_* = *Z_a_* + *λ* (*Z_a_* − *Z_ab_*), first draw a *λ* uniform based on a given Z_ab_.**Step 6.** The previous step should then be executed for every Z*_ab_*. The difference vector in a space with a-dimension of an is denoted by the notation (Z_a_ − Z_ab_), where a denotes the total number of variables present in the data.

ADASYN operates in a manner that is similar to SMOTE in that it produces synthetic observations for the minority class. However, it is based on adaptively generating additional samples for instances that are more difficult to learn, compared to those that are easier for the model. Similar to SMOTE, ADASYN produces synthetic observations in a single direction between a minority class observation and its k-nearest minority class neighbors. This line connects the minority class observations to the other minority class neighbors. The same number of k-nearest neighbors, 5, is selected for use with SMOTE. On the other hand, ADASYN produces a greater number of synthetic observations for minority class observations that contain a greater number of majority class observations within the region encompassing the k-nearest neighbors. In contrast, when a minority observation does not contain any majority observations within the scope of its k-nearest neighbors, no synthetic observations will be produced for this observation. This is because the majority of observations are required for the generation of synthetic observations. The justification for this can be found in the fact that learning from these observations is more difficult than learning from minority observations that are very different from the majority observations. This oversampling procedure is performed using ADASYN, Algorithm 3.
**Algorithm 3:** ADASYN**Require:** Choose the values q and *β*, which represent the number of nearest neighbors and the amount of class balance that should be desired after the synthetic data is generated. Consequently**Step 1.** The observation of majority and minority classes are represented by $g_{a}$ and $g_{b}$, respectively. R = ($g_{a}$
*−*
$g_{b}$) × *β*.**Step 2.** $F_{n}$ where n starts from 1 to $g_{b}$, which represent the category of minority observation, also a set of wholes $F_{n}\;$ is represented by L, and *L* ∋ $F_{n}$ for each $F_{n}$.**Step 3.** In order to determine $F_{n}$ of k-nearest neighbors, first it should compute the Euclidean distance that separates it from every other element in L.**Step 4.** k-nearest neighbor’s set of $F_{n}$ is represented by $G_{nq}$.**Step 5.** Define $\Delta_{n}$ as the observations number in the region bounded by $F_{n}$ of k-nearest neighbors that are members of the class that constitutes the majority. The ratio of *S_n_* can be computed as: $S_{n}=\frac{\Delta_{n}}{q},\;\;where\;n=1,\;\ldots ,\;g_{b}$.**Step 6.** $S_{n}$$\;\mathrm{can}\;\mathrm{be}\;\mathrm{normalized}\;\mathrm{based}\;\mathrm{on}\;\mathrm{the}\;{\hat{S}}_{a}\;$ = $\frac{S_{n}}{\sum^{g_{b}}{\;S}_{n}};$ thus, ${\hat{S}}_{a}\;$ is considered as a probability n  = $1\;\left(\sum_{n}{\hat{S}}_{1}=1\right).$**Step 7.** This is the number of observations that must be generated synthetically for every $F_{n}$; it can be computed as $P_{n}\;$= ${\hat{S}}_{a}\;\times \;R$.**Step 8.** Take a random instance of $P_{n}$ synthetic observations from $G_{nq}$ and designate them with the notation $F_{nm}$, where m can range from 1 to $P_{n}$.**Step 9.** Let us use the symbol *λ* to signify a number between 0 and 1. Generate a synthetic observation for a given $F_{nm}\;$ using the formula $w_{t}$ = $w_{i}$ + *λ* ($w_{i}$− $F_{nm}$), where it is drawn in the same way for each $w_{t}$. This will provide the synthetic observation. In this case, the difference vector in *n*-*dimensional* space is denoted by the expression ($w_{i}$ − $F_{nm}$), where *n* refers to the total number of variables present in the data. If *β* equals 1, then the approach will result in the dataset being perfectly balanced overall.

The effectiveness of the oversampling approaches known as SMOTE and ADASYN is analyzed and contrasted. A comparison is presented between the original dataset and the resampling dataset, using the SMOTE and ADASYN techniques. The comparison is carried out utilizing the Pima Indians with Diabetes dataset, three distinct classification models, and five distinct evaluation metrics, while utilizing three distinct preprocessing techniques for the data. To prevent data leakage, SMOTE and ADASYN were applied only to the training sets within each cross-validation fold, and the testing sets were not altered. According to the findings, the efficiency of the classifiers in the majority of instances is improved by using SMOTE and ADASYN. It was discovered that SVM, when combined with SMOTE, performed significantly better than ADASYN when the degree of class imbalance was increased. In addition, the SMOTE and ADASYN algorithms also have the effect of improving the relative performance of the random forest as the degree of class imbalance increases. Nevertheless, because the degree of class imbalance might vary from situation to situation, there is no preprocessing solution that reliably outperforms the others and contributes more to improved performance.

#### 3.2.4. Data Standardization (Feature Scaling)

As indicated previously, the majority of the available attributes in the Pima Indians with Diabetes and Frankfurt datasets are numerical. In addition, several of these characteristics were recorded using different measurement units. The effectiveness of the models could be negatively impacted if such features were processed without any normalization [45]. As a result, normalization is required in order to rescale all numeric attributes so that they fall into a range between zero and one. The process of rescaling features to achieve a standard normal distribution with zero mean and unit variance is known as standardization, which can also be expressed as Z-score normalization. As shown in Equation (3), the standardization process (R) also helps to ensure that the data distribution is less skewed.

(3) $$z=\frac{x-\mu }{\sigma }$$

where x represents the original feature value, μ is the mean of the feature, and σ is the standard deviation. On the other hand, many machine learning techniques, such as tree-based models, are undoubtedly examples of models for which feature standardization does not necessarily lead to substantial performance improvement.

### 3.3. Framework Training

This comprises the separation of the training sets from the test sets, the selection of the model to be used and the selection of the performance metrics to be used. After the preprocessing, the dataset is sufficiently prepared to be categorized in the next step. This step is followed by a feature-selection step based on the use of different techniques. The next step is to build models based on the training of different classification methods. Each technique has been trained using a prepared dataset-based preprocessing step. Therefore, this model includes the splitting of the dataset into training and testing, the selection of the most-relevant features, the selection of appropriate classifiers to apply to the dataset, and performance evaluation using cross-validation.

#### 3.3.1. Cross-Validation

Overfitting and underfitting are two of the most common challenges encountered in machine learning models. Overfitting occurs when a model learns the training data too well, resulting in a very low or zero training error. In this case, the model captures not only the underlying patterns but also the noise and variations present in the data. However, when the model is evaluated on unseen data, it fails to produce accurate predictions due to poor generalization. This occurs because the model has been overly tailored to the training data rather than the learning of generalizable patterns. The opposite of overfitting is underfitting, in which the model is unable to capture the underlying structure of either the training data or unseen data. Cross-validation is the solution that can help solve these problems. This step involves separating the data into two distinct sets: the training set, which is utilized for the purpose of model training; and the test set, which is utilized for the purpose of validating the model. It is possible to determine whether a model is overfitted by evaluating it on the test set, which contains data that has not been used during training.

**Figure 2:**
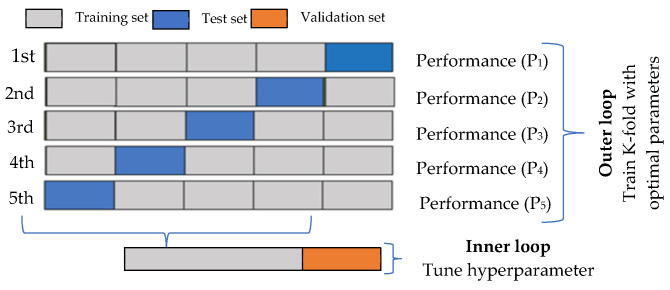
Hyperparameter tuning and training for a diabetic dataset based on KCV [47].

Figure 2 provides a visual representation of the 5-fold cross-validation method used in this research. K-fold cross-validation is a technique that is frequently utilized for model selection, error assessment of classifiers, and dataset partitioning. The dataset is split up into five different folds, and K−1 folds are used for training and the fine-tuning of the hyper-parameters in the inner loop, which is where the grid search method is implemented [46]. The assessment of the model is performed using the test data along with the optimal hyperparameter values during the k-th iteration of the outer loop. The raw dataset has an imbalanced class distribution of negative to positive samples; a stratified k-fold cross-validation (KCV) approach is applied to preserve the same percentage of samples belonging to each class as it was originally. The estimation of the overall performance is accomplished using Equation (6):

(4) $$X=\;\frac{1}{N}\sum_{m=1}^{m}y_{n}\pm \;\sqrt{\frac{{\sum_{m=1}^{n}\left(y_{m}-\;y^{-}\right)}^{2}}{N-1}}$$

where *X* is identified as the overall evaluation metric for the classifier, and *y_m_* ∈ *R m* = 1, 2, 3, … *N* is the performance metric for each fold in the classification process.

#### 3.3.2. Feature Selection

The process of selecting features is referred to as “variable selection” in the field of machine learning. It is used to select the relevant features that will be utilized in a system that is based on ML. It is important to choose the most relevant subset of predictors for a number of reasons, including the following: to run the ML-based system effectively and analyze the results; to prevent the curse of dimensionality; to reduce computational complexity and time; to decrease the amount of overfitting; and to enhance classification performance [3]. Our datasets contain a large number of features; if all of these features are used in training, the processing time and prediction complexity will increase. In addition, issues such as redundant features, irrelevant features, and the existence of a significant disparity among features can be addressed. In order to tackle all of these issues, a variety of methods are utilized in order to acquire a subset of the characteristics that are the most relevant to prediction difficulties [40].

Despite the large variety of available feature selection, oversampling, and boosting algorithms, the most representative and popular ones are used in the study, namely, GA, CFS, and RFE in feature selection; SMOTE in oversampling; and a soft-voting ensemble in boosting, to ensure that computational efficiency and interpretability are maintained. These selections are motivated by the demonstrated effectiveness of these algorithms in clinical data analysis and their ability to be integrated into the proposed hybrid model. The choice of these techniques was also aimed at achieving a balance between diversity of method and experimental transparency that would make it possible to control the effects of individuals and combined effects on predictive performance. In particular, the GA, CFS and RFE wrapper- and filter-based approaches provide complementary perspectives on feature relevance and optimization. Oversampling was used to balance the classes in the diabetes datasets, reducing bias for the majority of the dataset, although the dataset integrity functioned to preserve the integrity. This research systematically combined established and known methods to investigate their impact on predictive accuracy instead of considering all possible algorithms.

• Genetic Algorithm

The genetic algorithm is utilized in this research for the purpose of feature selection. A stochastic feature-selection approach is adopted because exhaustive search techniques are computationally expensive when applied to large sets of features. The population of candidate solutions is initialized by the algorithms; after this selection, crossover, mutation, and termination are carried out. The selection procedure is based on the concept of survival of the fittest. The formula for calculating the fitness function [48] is as follows:

(5) $$Fitness\;F(i)=\;\frac{fitness\;if\;indivitual\;F(x)}{sum\;of\;fitness\;of\;all\;indivituals\;F(X)}$$

Reproduction and genetic modification are the primary activities involved in the GA process. We utilized a single-point crossover method. To perform mutation, a bit value is randomly altered within the range of 1 to 7. Tournament selection was used because it provides a good balance between selection pressure and population diversity when selecting the individual who will become their offspring. The GA parameters were set in this study as follows: crossover rate of 0.85, mutation rate of 0.05, population size of 150, and number of iterations of 500. Algorithm 4 illustrates the process of GA for feature selection.
**Algorithm 4:** GAFS**Step 1.** Initialize: x equals to 0, the population individuals denoted as $N\left(x\right).$**Step 2.** Determine each individual’s fitness function within the population $N\left(x\right)$.**Step 3.** While there is a failure to meet the termination criterion.**Step 4.** Repeat x=x+1.**Step 5.** Individuals with a superior fitness level should be chosen based Selection.**Step 6.** Hybridization of the parent organisms to create new individuals-based Crossover.**Step 7.** Bit-flipping and other forms of transformation are made based Mutation.**Step 8.** Terminate the loops.**Step 9.** Return the healthiest members of the population as a whole.

• Correlation-based Feature Selection

By computing the inter-correlation values among various features, correlation-based feature selection determines the relevance of those features individually. In this approach, highly correlated and irrelevant features are eliminated to identify the features that are of the utmost importance. In addition, a variety of search techniques, such as the best-first search (BFS), the evolutionary search (ES), and other similar techniques, are utilized in conjunction with the correlation-based feature selection in order to explore important features [26]. Algorithm 5 illustrates the process of the CFS method.
**Algorithm 5:** CFS**Input:** The initial *m-dimensional* data, denoted by $p\;\in \;w^{m}$, and the anticipated result, denoted by p ∈q.**Output:** The data with a reduced number of *n-dimensions*, $q\;\in \;w^{n}$.**Step 1.** While g less than or equal to m repeat:**Step 2.** $xgy=\;\frac{\sum {(U}_{g}-\;U_{g}^{-})\;{(V}_{y}-\;V_{y}^{-})}{\sqrt{\sum {{(U}_{g}-\;U_{g}^{-})}^{2\;}}\times \sqrt{\sum {{(V}_{y}-\;V_{y}^{-})}^{2\;}}\;}$**Step 3.** In order to select the first k features for the $q\;\in \;w^{n}$ relationship, sort the correlation, xgy, by decreasing order.

• Recursive Feature Elimination (RFE)

The model operates within the framework of the wrapper technique to make certain that the most-relevant features are obtained as part of the data processing stage. Consequently, making use of this model is essential in order to detect features included within large or small datasets. RFE is a process that selects features based on the performance of the model. The metric evaluates the ability of individual features to differentiate one another. During each iteration, a ranking score is computed, and features with low importance are removed from consideration. The iterative technique is carried out in a loop until the required number of characteristics has been determined [40]. Obtaining feature weights that accurately reflect the relative significance of each feature is possible after a classifier has been trained with a training dataset. When all of the features are ranked according to their respective weight values, the one that is eliminated is the one that has the lowest weight value. The classifier is then retrained with the features that are still available until the desired subset of features is achieved. In conclusion, the overall ranking of the features can be determined by utilizing the RFE technique, which is based on the importance of the features [49].

#### 3.3.3. Classification-Based Machine Learning

The process through which a computing system learns the features of input data is referred to as machine learning. These approaches have been demonstrated to be successful in the diagnosis of diabetes. There are various types of machine learning algorithms, including supervised, unsupervised, and reinforcement learning techniques [4]. Because machine learning methods are data-driven, they have numerous practical applications. Because of the enormous volumes of data that are being entered into the database, machine learning has the potential to significantly reduce the amount of work performed by humans. These data are used to train models, which are then used to generate the output that is most appropriate given the input data. The models can be trained on any attainable parameter, taking into account both practical considerations and medical requirements. Some methods examine patients based on facial features, while others might examine the data gathered from clinical reports such as blood tests. Due to the existence of several symptoms of the disease, the criteria can vary significantly. Researchers have investigated many different algorithms and tuned numerous hyperparameters in order to produce the findings that appear to be most suitable for real-world applications.

• Multi-layer Perceptron (MLP)

A neural network is composed of processing units that are referred to as neurons, and each neuron is interconnected to other neurons in the network through weighted unidirectional connections. The MLP used in this study comprises multiple hidden input layers, and an output layer. The perceptron, which may be thought of as a linear combination of input and weights, serves as the fundamental component of this model. This work employs the ReLU activation function in the hidden layers and a sigmoid function in the output layer. Because there are eight variables involved, the input layer of the MLP architecture contains eight neurons. The intermediate layer is the hidden layer, and it is where the weights and input are responsible for the computation, utilizing a ReLU unit. The output layer is where the final prediction results are produced at the end [50]. Any layer of the MLP takes a D-dimensional input vector and produces an N-dimensional output vector, denoted by the function f(x): RD → RN. The end product of each processing unit can be represented mathematically, as in Equation (6).

(6) $$f(x)=\;\Phi \left(\sum_{j}w_{j}x_{j}+b\right)$$

where *xj* and *wj* are indicate the input and weights, respectively. Bias to the neuron is represented by Φ and the nonlinear activation function is represented by *b.*

Backpropagation is utilized to adjust the weights in order to reduce the error that occurs during class label prediction. During the training process, the parameters of the neurons are updated according to the formula in Equation (7), using back-propagation in order to reduce the amount of error, which is calculated as γ = *ytrue* − *youtput*.

(7) $$W_{new}=\;W_{old}+\eta \times \gamma$$

where η denotes the learning rate, which is the rate at which the weights are updated while the training is being done. Nevertheless, estimations of the number of hidden layers and neurons at each hidden layer are highly dependent on the dataset. The greater the number of layers and neurons, the greater the number of parameters, an increase which does not necessarily guarantee improved performance. In addition, the training dataset requires a proportionally larger number of samples as the number of parameters increases. The input data from the input layer is processed through the hidden layers using the initialized input values and weights. Each unit in the middle layer, which is referred to as the hidden layer, receives the net input, applies the activation function known as the ReLU activation function, and then turns the enormous amount of data into a more manageable range between 0 and 1. The calculation can be carried out for each of the middle layers. On the output layer a sigmoid activation function is applied, which ultimately results in the final output that contributes to the prediction associated with the diagnosis of diabetes.

The hyperparameters in this work, including the number of hidden layers, neurons in each layer, activation function, neuron initializer, batch size, learning rate, number of epochs, dropout rate, loss function and optimizer, are not hard-coded. They are instead optimized using a grid search approach to achieve optimal model performance on the provided dataset.

• SVM

A separating hyperplane serves as the formal definition of SVM, which is a non-probabilistic classification method. Based on the information that has been collected, the method generates and maximizes the margin from the support vectors in a supervised learning setting. The purpose of the SVM is to determine the optimal decision boundary in dividing an *n-dimensional* space into classes. This will allow new data points to be classified accurately. “Hyperplane” is the term used to describe this optimal decision boundary [51]. The SVM identifies the hyperplane that can most effectively differentiate between the two classes of instances. In two-dimensional space, this hyperplane can be interpreted as a line that divides the plane into two distinct categories. On the basis of the distance that exists between the two classes that it differentiates, it is possible to choose, from among the several hyperplanes, the one that is the best. The hyperplane that has the maximum margin between the two classes is referred to as the maximum-margin hyperplane. The key hyperparameters of the SVM classifier are the epsilon ε parameter, the regularization parameter, and the kernel parameter, while **ε is** typically associated with regression variants of SVM [39].

The data point of *n* can be defined as

(8) $$\left(\vec{a_{1}},\;{\;b}_{1}\right),\;\ldots ,\;\left(\vec{a_{x}},\;{\;b}_{1x}\right)$$

while *a*1 is the real vector, and *b*1, which can be either 1 or −1, represents the class to which *a*1 corresponds. It is possible to design a hyperplane in such a way as to maximize the distance between the two classes *b* = 1 and *b* = −1, which is described as the following:

(9) $$\vec{y}\;\cdot \;\vec{a}-z=0$$

where a normal vector is represented by $\vec{y}$ and the offset of the hyper-plane with $\vec{y}\;$ is indicated by $\frac{z}{\left\|\vec{y}\right\|}$.

The objective is to determine the hyperplane that provides the most effective division of the class. To evaluate which hyperplane is superior, the margin should be calculated, which is the distance that separates the planes from the data. A smaller margin increases the probability of misclassification, whereas a larger margin improves the model’s generalization ability. Therefore, the optimal hyperplane is the one that maximizes the margin. The margin is defined as the distance between the support vector of the positive and negative point, measured relative to the decision boundary.

• Random Forest

RF is one of the most widely used ensemble classification methods. It is composed of a large number of independent decision tree classifiers, in which each branch of the tree contributes a vote for the final prediction on test samples based on a predetermined set of criteria. This procedure can be described in the following manner [3,39]. The bootstrap method is a resampling technique used to extract certain samples from the training set to use as a training subset. After that, a certain quantity of features from the feature set for the training subset are selected at random to serve as the foundation for splitting each node of the decision tree. These steps will be repeated to generate multiple training subsets and corresponding decision trees, which will subsequently be combined in order to construct a random forest. During testing, the input samples are passed through the random forest, and then each decision tree produces a classification outcome. The final prediction is obtained through a majority voting mechanism in which the class receiving the highest number of votes is selected. This procedure is carried out on all test samples until the test set has been classified.

### 3.4. Model Diagnosis

**Figure 3:**
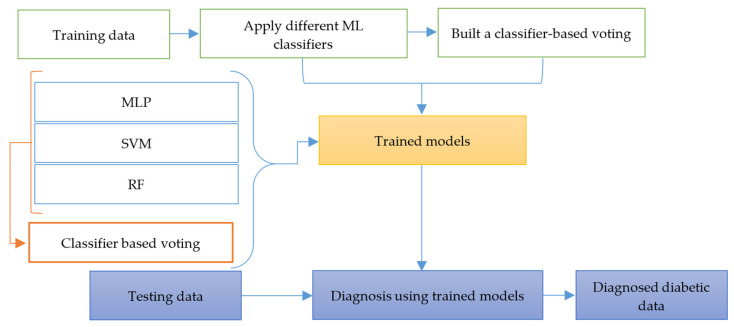
Training and testing model for diabetic diagnosis. The arrows indicate the flow of data through the different stages of the proposed model, from input data processing to classification and final prediction.

The classification and prediction of diabetic disease are the primary goals of the proposed method. Figure 1 provides a high-level overview of the various ML-based systems presented in this study. Figure 3 depicts the training–test set paradigm commonly used in machine learning systems. The first step is to split the dataset up into two different subsets, namely the training set and the test set. The diagnosis of diabetes is carried out based on the outputs of the trained learning model. To achieve this, the dataset was initially gathered and integrated and then processed for further analysis. Second, different techniques were utilized for data preprocessing. Subsequently, feature-selection methods, including correlation, RFE, and GA, were employed to reduce the number of features. Finally, several classification algorithms for diabetes were employed.

The MLP, SVM, and RF algorithms are used for this purpose. The proposed MSR framework is designed by combining three classifiers to reduce prediction variance and enhance stability by aggregating probabilistic outputs from multiple classifiers using soft voting, rather than relying on a single decision boundary. The parameters of the machine learning algorithms trained on the diabetic dataset are presented in Table 3. In addition, a voting-based ensemble classifier has been developed by combining the outputs of the individual base classifiers. Since it incorporates the findings from multiple models, the voting classifier generally produces more robust, and on average, more accurate, results relative to the individual models. SVM, MLP, and RF classifiers generate an independent prediction, which is then aggregated using a voting mechanism. The final prediction is obtained through majority voting. Figure 2 provides a visual representation of the proposed algorithm.

The hyperparameters presented in Table 3 were used to train each model independently. In the case of ensemble predictions, because of the need to make predictions for each class, the class probabilities of all the classifiers were employed. No additional probability calibration was applied, since all base models normally give reasonably calibrated probabilities. The voting weights were determined empirically using cross-validation on the training set; classifiers with better performance were assigned slightly higher weights. In the case where the differences in performance were insignificant, equal weights were used instead. This approach enables MSR to capitalize on the complementary advantages of the base models and enhance general predictive performance.

All experiments for the different categorization approaches were conducted. The dataset was divided into two subsets: training and testing. The models were trained using a 5-fold cross-validation scheme, which also had been incorporated into the training and testing phases of the process. All the preprocessing steps (imputation, oversampling, standardization and feature selection) were applied only to the training data within each cross-validation fold and then subsequently applied to the corresponding test data.

The hyperparameters shown in Table 3 were selected using a grid search over a predefined parameter space to achieve optimal model performance. For the MLP classifier, we tested the size of hidden layers [16, 32, 64], the learning rate [0.001, 0.01, 0.1], the alpha [1 × 10^−5^, 1 × 10^−4^, 1 × 10^−3^], and momentum [0.1, 0.2, 0.5]. For the SVM classifier, the penalty C [0.1, 1, 10], gamma [‘scale’, ‘auto’] and kernels [RBF, linear] were tried. In the case of the random forest classifier, the following parameters were tested: estimators [100, 200, 300], min-samples-split [2, 5, 10] and criterion [gini, entropy]. Feature selection was performed independently within each cross-validation fold using recursive feature elimination (RFE), based only on the training data. This procedure ensures that no information leakage occurs from the test set and enables an unbiased evaluation of model performance.

Third, all training, prediction, and evaluation process are conducted in an iterative manner to establish all relevant performance measurements for each classifier. For each classifier under the cross-validation strategy, the arithmetic mean of each accuracy number is calculated. The classifiers are then ranked based on their accuracy values for each classifier, which are arranged in descending order. The final step is to determine the candidate classifier, which is used to diagnose diabetes patients based on the ranks of all classifiers. The candidate model will then be proposed for a particular diabetic diagnosis task when this step is complete. Finally, the effectiveness of the classifiers is assessed using six performance metrics, namely ACC, SE, PPV, NPV, FM, and AUC.

### 3.5. Data Leakage Control and Evaluation Integrity

To avoid information leakage and to maintain the validity of the reported performance, all data-dependent operations, such as imputation of missing values, over-sampling (SMOTE), feature scaling and feature selection were strictly performed within each training fold during cross-validation.

In particular, preprocessing and feature-selection models were trained only on the training subsets, and the learned transformations were applied on the respective validation subset. No validation or test samples were used at any point in preprocessing, feature selection or model training. This fold-wise preprocessing strategy guarantees that there is no overlap between the training and evaluation data and that no information leakage occurs. The strong classification performance observed in this study is attributed to the combination of hybrid feature selection, class imbalance handling, and ensemble learning, rather than any data leakage.

Even though the performance is high, the results were obtained with the help of strict cross-validation using leakage-free preprocessing. As a result, the results are to be used as the demonstration of methodological effectiveness when within the conditions of controlled evaluation, not as ultimate signs of clinical deployment performance.

To further address overfitting concerns arising from the multi-component framework, the contribution of each component (imputation, oversampling, feature selection, and ensemble learning) was analyzed in terms of the training–validation performance gap across cross-validation folds. The training and validation metrics were continuously monitored to ensure that there was no significant deviation during model development. The proposed MSR framework was designed to enhance generalization by combining the predictions of multiple base classifiers and thus causes reductions in variance and the effects of the possible overfitting of individual models. Experimental results demonstrate that the proposed ensemble achieves a more consistent and stable performance than those of the individual classifiers, indicating better generalization ability, rather than a simple averaging of overfitted models.

### 3.6. Additional Validation and Robustness Analysis

In an additional attempt to mitigate the risks of overfitting and over-optimistic performance, further validation experiments were conducted. Specifically, repeated stratified k-fold cross-validation was employed to test the proposed model, ensuring robustness across different data partitions. In addition, the consistency of the performance was measured by evaluating the differences in evaluation metrics across the folds. The low variance observed in accuracy, sensitivity, and AUC values indicates that the model performance is not sensitive to a particular data split. Furthermore, the ROC curves were generated using predictions which were obtained only from unseen validation folds, while no training data were used for their computation. The high values for AUC demonstrate high class separability due to the selected feature subsets, rather than data leakage or memorization.

To further validate the generalization capability, the performance of the model was also compared with the variations in feature-selection combinations and classifiers. The consistent ranking of classifiers across all configurations indicates that the observed performance is not dependent on a specific preprocessing pipeline, but rather reflects the inherent discriminative capability of the model. Lastly, it is admitted that the assessment was performed using benchmark datasets, and despite the high level of cross-validation processes, external validation using independent datasets is recommended for future work in order to provide more evidence regarding the applicability of the suggested method.

## 4. Experimental Results

The purpose of this section is to evaluate the efficacy of both the proposed model and the state-of-the-art approaches. During the development of the proposed model, multiple phases were performed to improve the effectiveness of the proposed model, and three ML approaches, namely, MLP, SVM, and RF, were trained and evaluated. The 5-fold cross-validation was used to obtain all the reported results and performance met-metrics are the average of folds. All experiments were conducted on the Python 3.7 development platform using a workstation with the following specifications: a computer system equipped with a 13th Gen Intel^®^ Core™ i7-1355U processor (Intel Corporation, Santa Clara, CA, USA) and 32 GB RAM, running a 64-bit operating system.

The time and space complexity of the proposed pipeline was tested using the Pima Indians with Diabetes, Frankfurt, and combined datasets. The preprocessing stage was computationally efficient, requiring less than 30 s for the largest dataset. In contrast, GA-based feature selection was the most computationally expensive step, requiring approximately 800–1000 s on the combined dataset. RFE and CFS were quicker, and hybrid combinations required longer execution times due to sequential processing. Classifier training and the proposed ensemble of MSR introduced moderate computational overhead (100–150 s), whereas inference was highly efficient, taking less than 5 s. The highest memory consumption was dependent on dataset size, peaking at around 1.5 GB for the combined dataset in ensemble training. Those findings indicate that the framework is both computationally efficient and scalable, as well as the fact that it has high predictive performance (98.0% accuracy, 97.43% sensitivity, 99.03% specificity, 99.51% precision, and 98.72% F1-score).

The assessment plan is based upon a rigorous two-step validation plan to ensure unbiased performance estimation. First, the data is divided into independent training and test subsets, where the test set remains completely unseen during model development. Second, 5-fold cross-validation is applied only within the training set, hyperparameter optimization, feature selection, and model selection. All preprocessing operations are performed exclusively on the training folds.

Once the model has been selected, the final trained model is tested on an independent test set that has not been used at any stage of training, validation, preprocessing, or model selection. All performance metrics reported in this study rely on the test on this untouched test set, ensuring unbiased and reproducible performance evaluation.

The efficacy of the generated models has been evaluated consistently using several assessment metrics, including accuracy, sensitivity, specificity, and precision, as well as the F1-score. Accuracy refers to the proportion of correctly predicted instances relative to the total number of instances in the testing phase. The term sensitivity provides information regarding the percentage of “true positives” that are accurately classified when the test is being performed. The percentage of true negatives that are accurately identified by the test is indicated by the parameter known as specificity. The percentage of occurrences that a classifier has categorized as positive in comparison to the overall number of positive predictions is referred to as the precision. The F1-score provides an indication of the harmonic mean of precision and recall [45].

(10) $$Acc=\frac{\left(TP+TN\right)}{\left(TP+TN+FP+FN\right)}\times 100\%$$

(11) $$Sens=\frac{TP}{\left(TP+FN\right)}\times 100\%$$

(12) $$Spec=\frac{TN}{\left(TN+FP\right)}\times 100\%$$

(13) $$Pre=\frac{TP}{\left(TP+FP\right)}\times 100\%$$

(14) $$F1\text{-}score=\frac{2\times TP}{\left(2\times TP+FN+FP\right)}\times 100\%$$

If the actual value of the target in the dataset is true and the classifier correctly predicts that it will be true, then the prediction is a true positive (TP). On the other hand, if the classifier predicts it as negative, then the prediction will be a false negative (FN). Similarly, if the actual value of the target is false and the classifier predicts it as such, then the prediction is true negative (TN). On the other hand, if the classifier determines that it will be true, then the prediction will be false positive (FP).

### 4.1. Performance Evaluation-Based Oversampling Methods

The summary of both datasets is presented in Table 4. Because number of negative instances is higher than the number of positive instances, as shown in Table 4, there is an imbalance between the classes. This is because there are more negative values than positive ones. To address this issue, different oversampling techniques have been carried out; rather than focusing solely on accuracy, other evaluation metrics are also considered, such as precision, specificity, sensitivity, and F1 score. Because both sets of data consist of female patients, the number of pregnancies is one of the important characteristics. Furthermore, to determine whether or not a patient has diabetes, three machine learning classifiers are evaluated on the aforementioned datasets and the results are analyzed.

It was found that the two oversampling algorithms, SMOTE, and ADASYN, resulted in different performance outcomes on the Pima Indians with Diabetes imbalanced dataset in three classifiers. Evaluation on multiple metrics provided a comprehensive overview of classifier performance under different preprocessing settings (see Table 5).

Based on these findings, the performance of the MLP and RF classifiers improved significantly, and was enhanced significantly because of oversampling. In the case of MLP, the highest sensitivity (84.63%) and F1-score (85.02%) were achieved using SMOTE, whereas ADASYN also improved performance, yielding a sensitivity of 79.96% and F1-score of 79.51%, compared to the original dataset. Similarly, RF performed better with oversampling with the best performance, with 90.91% and 91.04%, in terms of sensitivity and F1-score, respectively, with SMOTE and ADASYN. These findings suggest that oversampling is effective in mitigating the impact of class imbalance for both MLP and RF. Conversely, the performance of SVM remained relatively stable across different preprocessing. Slight decreases in sensitivity were observed with SMOTE (75.87%) and ADASYN (75.98%), compared to the original dataset (77.03%), with the F1-scores also indicating a slight decline. This implies that in this case, oversampling does not have a significant positive effect on SVM and can also result in a minimal decrease in sensitivity. Overall, MOTE appears to be slightly more effective than ADASYN for both MLP and RF in terms of sensitivity and F1-score. However, no single preprocessing method consistently improves performance across all classifiers, indicating that the effectiveness of oversampling is classifier-dependent. These results highlight the importance of selecting appropriate preprocessing strategies when dealing with imbalanced data, in order to achieve balanced classifier performance.

### 4.2. Performance Evaluation and Analysis

In this investigation, several preprocessing procedures were implemented, including feature scaling, two data oversampling strategies, and missing data imputation. In addition, the proposed framework integrates three feature-selection methods, three base classifiers, and a voting-based ensemble model.

**Figure 4:**
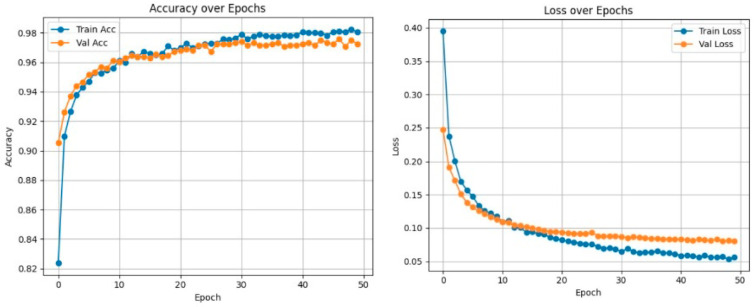
Training and validation accuracy and loss curves showing smooth convergence, stable learning, and good generalization without overfitting.

Epoch-wise accuracy and loss curves were used to evaluate the training dynamics of the proposed model. As shown in Figure 4, both training and validation accuracy increase steadily with minimal variance, while the loss decreases consistently over epochs. The validation curves do not have any significant variations and the training and validation performance indicate strong generalization and the absence of overfitting. The curves stabilized after some epochs, demonstrating convergence and effective learning of underlying patterns rather than memorization. These findings support the strength and consistency of the proposed strategy. Additionally, the performance of the proposed model has been compared with standard baseline models based on standard evaluation metrics to provide a comprehensive assessment of its effectiveness.

Table 6 presents the classification results of the Pima Indians with Diabetes dataset by using three feature-selection algorithms, namely, GA, correlation-based feature selection (CFS), and RFE, and four classifiers, namely, MLP, SVM, RF, and the voting ensemble associated with MSR. The findings indicate that feature selection significantly improves in the predictive performance of all the classifiers. The RFE-based feature subset produced the best overall performance, and was closely followed by GA, and CFS offered moderate improvement. In particular, the RFE with the MSR voting classifier achieved the highest performance, 89.91%, sensitivity of 90.11%, specificity of 89.22%, precision of 91.37% and F1-score of 92.87%. The GA with MSR also had good results, with an accuracy of 88.72%, meaning that it demonstrates effectiveness in capturing nonlinear feature relationships that can be used in the prediction of diabetes. Conversely, CFS-based features reported lower accuracy (85.41%) with MSR, suggesting that simple correlation-based technique is less effective than iterative or evolutionary feature-selection technique.

In all feature-selection methods, the MSR voting classifier consistently outperformed the single models, which demonstrates that ensemble learning can effectively integrate to synthesize the advantages of different classifiers in order to reach greater stability and generalization. Random forest was the highest among the individual classifiers, with an 87.22% accuracy with RFE, which reiterates the fact that it is able to model nonlinear relationships that are complex nonlinear relationships. SVM exhibited competitive and stable performance in all the methods, which suggests its efficiency in the processing of data that is linearly separated, whereas MLP had a slight decrease on accuracy, but displayed balanced sensitivity and specificity, which could suggest underlying patterns in biomedical data capture.

The fact that the results have improved proves that feature selection is a very important aspect in improving classification performance. GA was able to achieve an accuracy of 3–5%, compared to the baseline, without feature selection, and RFE was able to achieve a result of 4% over GA, since it is effective at finding the most discriminative features. CFS was computationally efficient even though it did not yield substantial performance gains. Altogether, RFE and the MSR voting ensemble performed the best trade-off across all evaluation metrics, proving it to be the optimal configuration for obtaining reliable diabetes classification. As such, this setting, as the most successful one, was used in later comparative studies with the Frankfurt and mixed datasets.

The analysis of the Frankfurt dataset performance demonstrates that feature selection improves classification performance across all classifiers (see Table 7). The features using RFE methods consistently achieved the best performance, followed closely by GA, and correlation-based selection provided moderate improvements. Random forest was the strongest performer among individual classifiers, with a high of 86.14% accuracy using RFE, and SVM exhibited stable performance relative to the feature selections. MLP had slightly lower accuracy and equal sensitivity and specificity. The MSR voting classifier consistently outperformed all individual models, with RFE + MSR recording the highest accuracy (89.38%), sensitivity of 90.21%, specificity of 88.74%, precision of 91.12% and F1-score of 90.67%. These findings support the claim that using the MSR ensemble of multiple classifiers achieves improved generalization and stability even though iterative feature-selection algorithms such as RFE offer the most discriminating features in the prediction of diabetes.

In the combined dataset, it is observed that feature selection and the MSR voting ensemble improve predictive performance with all the classifiers (see Table 8). The most discriminative feature subset was consistently identified by RFE, and this had the highest accuracy (89.91%) with MSR. GA and correlation-based feature selection were also found to be better than baseline, though RFE produced the best trade-offs between accuracy, sensitivity, specificity, precision and F1-score. Random forest and SVM were found to have comparable performance, with MLP being slightly less accurate. MSR voting classifier consistently outperformed single models, which supported the determination of the benefit of ensemble learning in diabetes prediction. Overall, the usage of RFE with the MSR ensemble was found to be the most effective configuration with the combined dataset.

**Figure 5:**
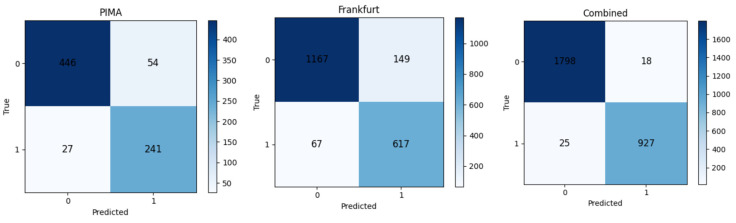
Confusion matrices of the best-performing configuration (RFE + CFS + SVM) on the Pima, Frankfurt, and Combined datasets.

To enhance clinical interpretability, confusion matrices of the best-performing configuration (RFE + CFS + SVM) were generated for each dataset. The proposed approach has a balanced distribution between the true positives and true negatives, and the rates of false negatives (FN) and false positives (FP) are low rates, as presented in Figure 5.

The interpretation of classification errors, particularly FN and FP, is important in medical diagnosis. False negative occurs when a patient with diabetes is incorrectly classified as non-diabetic, which is clinically serious since it may lead to delayed diagnosis and severe complications, including cardiovascular diseases, neuropathy, kidney failure and vision loss. Therefore, the reduction of FN is crucial in the early-stage reliable screening.

Conversely, a false positive is the case where a non-diabetic person is incorrectly classified as diabetic. Although less severe than an FN, FP cases can lead to unnecessary diagnostic tests, higher healthcare expenses, and psychological stress among patients. In the most effective configuration used, the proposed model shows high sensitivity (up to 97.43) and specificity (up to 99.03), which means that both the false negative and the false positive rates are significantly low. The high sensitivity shows that the model is effective in correctly identifying diabetic patients, and thus it minimizes the chances of false diagnosis, as the high specificity means that false alarms are minimal.

Clinically, the results infer that the proposed hybrid model can be effectively applied in early-stage diabetes screening at an early stage where the primary goal is to correctly detect the actual cases rather than prevent all false positives. The balance between sensitivity and specificity highlights the potential of the model in supporting reliable clinical decision-making.

### 4.3. Feature-Based Performance

Table 9 presents the average performance of four classifiers, MLP, RF, SVM, and MSR, before and after the implementation of four feature-selection methods.

The findings reveal that feature selection has a positive impact on the performance of the classifier. In the absence of feature selection, the classifiers achieved an average accuracy of 85.77%, and the sensitivity (83.81%) and F1-score (83.00%) were relatively lower. Using the Correlation technique slightly enhanced the overall performance, as the average accuracy and F1-score improved to 87.78% and 85.20%, respectively. Further improvement was seen in the RFE, which attained an average accuracy of 89.29%, and which, in comparison with the sensitivity (88.69%) and specificity (88.71%), demonstrated a more balanced performance in classification. GA had the best average results over the four classifiers, with an accuracy of 91.04%, sensitivity of 91.42% and F1-score of 91.37%. This shows that GA performed best in the process of choosing informative features, which thereby resulted in better classifier generalization. These findings indicate that the feature selection significantly enhances classification performance, with GA providing the best average performance on this dataset.

The findings demonstrate that hybrid feature selection is generally more effective than single feature-selection methods, which implies that global search (GA) with redundancy-conscious (CFS) or wrapper-based (RFE) techniques produces a more discriminative feature subset. This results in corresponding improvements in both sensitivity and specificity, which is essential for reliable early-stage screening of diabetes. The performance of the classifiers was evaluated by considering three integrated feature-selection techniques, namely, GA + RFE, GA + Correlation, and RFE + Correlation. The performance of each classifier and the analyses of ROC curves for each integrated feature-selection method were computed and analyzed.

The SVM classifier-based GA+RFE feature-selection method achieved the best performance (see Table 10), having an accuracy of 98.2%, sensitivity of 97.03%, specificity of 99.37%, precision of 99.07% and F1-score of 98.26%, indicating its strong ability to correctly classify both positive and negative examples with strong precision. The MSR voting classifier was also competitive, with an accuracy of 97.1% and balanced sensitivity (96.43%) and specificity (98.11%), which indicates the effectiveness of ensemble-based prediction. The sensitivity of the MLP classifier was slightly lower, at 94.73%, though specificity (97.54%) and precision (97.05%) were high, which indicated good overall performance. Meanwhile, the RF classifier had the lowest accuracy (95.8%), but also an acceptable level of sensitivity (95.21%) and specificity (96.22%). On the whole, the GA + RFE approach benefits all classifiers, especially SVM and MSR, as it can choose informative and discriminative features and, as a result, improve classification performance.

In the case of GA + CFS feature-selection method, the SVM classifier again achieved the best performance, although one slightly lower than in the case of GA + RFE, and the results are as follows: accuracy of 97.6%, sensitivity of 96.71%, specificity of 98.62%, precision of 98.71%, and F1-score of 97.07%. The MSR voting classifier had a strong performance, with an accuracy of 96.5%, and balanced sensitivity (95.13%) and specificity (97.06%), which highlights the effectiveness of the ensemble-based prediction. The MLP classifier showed a slight decrease in sensitivity (93.23%) and accuracy (95.5%), while the performance of the RF classifier, which had the lowest performance, with an accuracy of 94.9%, sensitivity of 94.66% and specificity of 95.73%, suggested some sensitivity to feature reduction due to correlation filtering. Overall, these findings suggest that GA + CFS offers a viable feature subset, although it may result in a slight reduction in predictive performance for certain classifiers, compared to GA + RFE (see Table 11).

In the RFE + CFS feature selection, the SVM classifier achieved the highest performance, with an accuracy of 98.0%, sensitivity of 97.43%, specificity of 99.03%, precision of 99.51% and F1-score of 98.72%, results which are comparable to the GA + RFE results, as indicated in Table 12. This implies that a combination of RFE and correlation-based filtering is effective in preserving informative features. The MSR voting classifier also demonstrated strong performance, with an accuracy of 97.0% and balanced sensitivity (96.12%) and specificity (97.18%), reflecting the stability of the ensemble predictions. The MLP and RF classifiers showed moderate performance, with accuracy values of 95.9% and 95.3%, respectively, which shows that these classifiers are more sensitive to the feature-selection method. In general, the findings indicate that RFE with correlation-based filtering offers a strong feature subset, especially as to those features that are useful in the SVM and MSR classifier and have modest impacts on the performance of MLP and RF.

In general, SVM consistently achieved the best performance in all feature-selection methods and showed superior capability in both positive and negative class identification, as well as balanced performance measures. Another criterion that supports the effectiveness of ensemble-based methods is that the MSR voting classifier showed a consistent performance, regardless of the method. Although, for the most part, MLP and RF classifiers generally performed well, their performance was more sensitive to the selection of the feature-selection method, which was evident in slight reductions in accuracy and sensitivity under GA + CFS and RFE + CFS. GA + RFE and RFE + CFS presented the most informative and discriminative feature subsets, and resulted in the best overall performance relative to the classifiers, but GA + CFS resulted in small decreases in sensitivity and accuracy on some classifiers. These results support the role of proper feature-selection strategies in maintaining balanced and reliable performance, especially in datasets with possible class imbalance, and point to the fact that ensemble or robust classifiers such as SVM and MSR are most likely to gain the benefits of optimized feature subsets.

**Figure 6:**
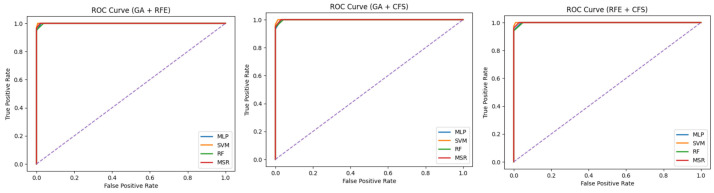
ROC curves for all classifiers based combined feature-selection methods.

The Receiver Operating Characteristic (ROC) curves for each classifier under the hybrid feature-selection frameworks are presented in Figure 6. The ROC curves reflect the rate of the true positive (TPR) and false positive (FPR) which offers a thorough assessment of the discriminative power of a classifier beyond the conventional performance measures. As can be seen, all the classifiers show good results; curves are near the upper-left corners, signifying high sensitivity as well as low false positive rates. The SVM classifier was the most successful model evaluated, with the steepest ROC curve and highest area under the curve (AUC), and the next successful were the MSR ensemble model and the RF model. The MLP classifier performance showed comparatively lower performance, as indicated by a relatively low curve. The excellent performance demonstrated by the ROC curves, despite reservations to the contrary, was only created using unseen test data in each fold of the cross-validation. Therefore, the observed strong ROC characteristics reflect high feature discriminability rather than overfitting or data leakage.

Among the feature-selection methods, GA + RFE produces the most distinct and well-defined ROC curve, indicating superior feature discrimination and improved class separability. The performance of the GA + CFS and RFE + CFS methods is also high but the curves are slightly less steep, indicating that there is a small trade-off between the sensitivity and specificity. Overall, these findings support the conclusion that hybrid feature selection significantly enhances in the discriminative capability of all the classifiers.

**Figure 7:**
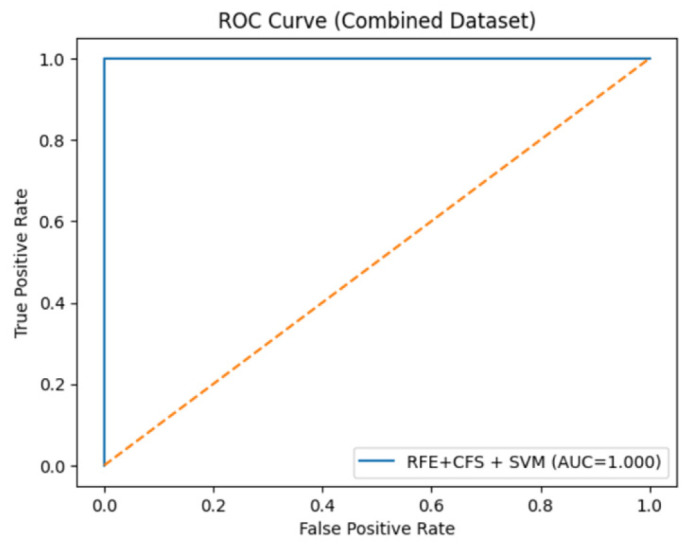
ROC curves of the proposed RFE + CFS + SVM model on the combined datasets.

Figure 7 illustrates the ROC curve of the best-performing configuration, RFE + CFS + SVM, on the combined dataset. The curve is located close to the upper-left boundary, which indicates near-perfect classification performance. The respective value of AUC (e.g., 0.98) indicates a high level in separating classes and demonstrates the stability of the suggested model. These high values of the AUCs show that the model is effective in distinguishing diabetic and non-diabetic cases. More significantly, the ROC curves were plotted based on unseen test data which had not been involved in any training, preprocessing, or model selection. Moreover, all the preprocessing operations were done in cross-validation folds to avoid the data leakage risk. The model was also checked via the 5-fold cross-validation and the results indicated low variance between the folds, indicating that the model is stable and highly generalized. The agreement in the performance between cross-validation folds further suggests that the model does not overfit to specific data partitions. Consequently, the ROC behavior that is observed is indicative of true learning of discriminative patterns as opposed to memorizing. This proves the validity and strength of the suggested framework in making clinical choices in diabetes prediction. The cross-validation consistency of performance across the folding of the validation set also suggests that the model is not overfitting on particular data splits, as seen by the fact that the fold variance in metrics of evaluation is low.

### 4.4. G-Mean Analysis of Classifiers

The balanced score of the classifiers, used to determine the performance in distinguishing the positive and negative classes, was calculated using the G-Mean metric. This metric is particularly important in datasets that may have a disproportionate number of classes. The formula of G-Mean is defined as the geometric mean, as expressed in Equation (15) [52]. It provides a single measure that reflects the classifier’s ability to correctly identify positive instances while also correctly rejecting negative instances. Table 13 presents the G-Mean of each classifier under the combined methods of feature selection (GA + RFE, GA + Correlation, and RFE + Correlation). Among the classifiers, SVM and the MSR voting ensemble consistently achieved the highest G-Mean values, indicating a well-balanced performance in terms of sensitivity and specificity. The MLP and RF classifiers also demonstrated satisfactory performance, although with slightly lower G-Mean values.

(15) $$G\text{-}Mean=\sqrt{Sensitivity\times Specificity}$$

As an illustration, the SVM and MSR classifiers under the GA+RFE feature-selection method achieved a G-Mean of 0.98, which is strong balanced performance across both positive and negative classes. In contrast, the MLP classifier had a G-Mean of 0.96, whereas RF had 0.955, reflecting a slightly lower but still strong balanced accuracy. In other feature-selection techniques, SVM and MSR were always at the top of G-Mean value, which demonstrates their reliability and robustness and consistency in handling class imbalance.

The ablation study was conducted to quantify the contribution of each pipeline component by comparing the preprocessing stages (imputation, oversampling, and standardization), single feature selectors (GA, CFS, RFE), hybrid feature selectors (GA + CFS, GA + RFE, and RFE + CFS), and the MSR ensemble with individual classifiers (MLP, SVM, and RF).

To determine the contribution of each component of the proposed pipeline (Table 14), we performed a partial ablation study. The table shows gradual improvements across preprocessing (imputation, oversampling, and standardization), single feature selectors (GA, CFS and RFE), hybrid feature selectors (GA + CFS, GA + RFE, RFE + CFS, and MSR ensemble). The findings show that the selection of hybrid features, particularly when combined with SVM and the ensemble of the MSR, contributes most significantly to predictive performance. Although the results follow the expected trend, future work should include a more detailed ablation analysis by independently evaluating each preprocessing step and feature-selection method to provide a more comprehensive assessment.

### 4.5. Performance Comparison

This study differs from most previous work, in which researchers normally use one feature-selection algorithm or test the algorithm on a single dataset. In contrast, it demonstrates that the hybrid feature-selection algorithm with an MSR ensemble has a strong and generalizable performance over a variety of datasets. Table 15 provides the comparison of the different machine learning approaches used to predict diabetes in the different datasets, including Pima Indians with Diabetes, NHANES, Frankfurt, and the combination of these datasets. Previous researchers using the Pima Indians with Diabetes data had moderate to high accuracies, with values ranging from 76.8% (Logistic Regression [53]) to 96.27% (DCSGAN [54]). The conventional classifiers KNN [55,56], RF [57], SVM [14] and neural networks [13] usually had accuracies between 77% and 95%. Although these models provided reasonable predictive performance, they often suffered from class imbalance issues and limitations in identifying the most informative features. In contrast, methods employing data augmentation or hybrid methods, including TF-KNN [25] and deep learning [1], were more sensitive and had better F1-scores, which means they were more effective at handling the minority classes. Recent reports on hybrid and ensemble approaches indicate that they have been demonstrated to perform better. For instance, the DCSGAN [54] and Multi-Layer Neural Network [58] on the Pima Indians with Diabetes dataset reached a 96.97% accuracy. The Pima Indians with Diabetes dataset integration with the Frankfurt datasets provided an ensemble method [59] which achieved 98.79% accuracy, and this illustrates the advantages of using many datasets to enhance generalization. These findings suggest that model robustness can be enhanced by integrating diverse data sources and ensemble strategies, thereby reducing overfitting.

Feature selection plays a critical role in this study. Hybrid techniques, such as GA + RFE, GA + CFS, and RFE + CFS effectively identify the most informative features, reduce dimensionality, and improve class discriminability. RFE + CFS + SVM provides the highest performance for the combined dataset, with 98.0%, 97.43%, 99.03%, 99.51% and 98.72% accuracy, sensitivity, specificity, precision, and F1-score, respectively. Likewise, GA + RFE + SVM attained 98.2% accuracy at the same sensitivity and specificity, which indicates the strength of hybrid feature selection and SVM. Across all datasets, SVM consistently outperformed MLP and RF, likely due to its ability to handle high-dimensional data and complex decision boundaries. The proposed MSR ensemble, which integrates a number of classifiers using soft voting, also achieved strong performance on single datasets (Pima Indians with Diabetes and Frankfurt). Nevertheless, its performance on the combined dataset was marginally worse than the optimized hybrid SVM settings, indicating that as much as the ensembles are capable of stability improvements, the selective choice of features and hyperparameter optimization is essential in the attainment of optimal accuracy.

The findings indicate that the hybrid feature selection outperforms single-method approaches and significantly enhances model performance. Hybrid selection is particularly effective for SVM because this method is sensitive to the relevant features and the dataset combination enhances the generalization, whereas optimized models need to balance sensitivity and specificity. The ensemble models are robust, but well-optimized single classifiers can be used with hybrid feature selection for better performance. The proposed hybrid feature selection with SVM provides a state-of-the-art feature-selection framework, one which is able to predict diabetes in its early stages with a high accuracy and with balanced sensitivity and specificity, and at the same time capable of achieving high F1-scores on both the single datasets and the combined data. This confirms the significance of preprocessing, feature selection and optimization of the classifier for effective medical diagnosis.

### 4.6. Discussion

This paper addresses the problem of early diabetes prediction on two benchmark datasets (Pima Indians with Diabetes and Frankfurt) and the combined data. The primary objective is to develop a robust and generalizable framework by integrating preprocessing, feature selection, and classification techniques. Oversampling, standardization, imputation and data cleaning were preprocessing steps that ensured high-quality input data. These steps were essential, because in diabetes datasets, missing or imbalanced data may exist, and this can negatively affect the performance of the classifier. Statistical procedures indicate that preprocessing improves the distribution and quality of the data, providing a solid foundation towards downstream modeling. The preprocessing of the data, such as oversampling and selection of features, was strictly applied only to the training data within each cross-validation fold; otherwise, there was the risk of some leakage of information into the test set. This ensures that the reported results reflect the actual model learning and not memorization.

The method of feature selection was crucial in improving the performance of the classifiers. Three separate feature-selection methods (GA, CFS, and RFE), and combinations of the three were tested, as well as three combinations utilizing these three (GA + CFS, GA + RFE, and RFE + CFS). Hybrid feature selection consistently outperformed the single methods, enabling the identification of the most discriminative and informative features, and dimensionality reduction with no loss of the relevant information. RFE + CFS + SVM and GA + RFE +SVM were also found to be the best combinations, with the highest accuracy, 98.0% and 98.2%, respectively, when using the combined dataset with balanced sensitivity, specificity, precision, and F1-score. This confirms that feature selection is very important in ensuring state-of the art predictive performance. The results of the classifier analysis demonstrated that SVM was always better than MLP and RF in all datasets and feature-selection techniques. This advantage can be explained by the efficiency of SVM in the high-dimensional space and its capability in representing complicated decision boundaries. The MSR soft voting ensemble also demonstrated strong performance, especially in single datasets, where its predictions were stable and demonstrated robust performance. Nevertheless, on the combined data, optimized SVM settings slightly outperformed MSR, which suggests that combination techniques are most effective when applied together with the attentive feature selection and hyperparameter optimization. The fact that the performance of the proposed model was similar across cross-validation folds is also another indicator that the model is strong and can effectively generalize without overfitting the data on particular data splits. This consistency across folds also shows that the model is not fitting to certain data splits and that it can be applied well to unseen data.

The comparative stability of the performance of various feature-selection and classification settings (such as GA + RFE + SVM, RFE + CFS + SVM and MSR ensemble) can be explained by the structured nature of the datasets and the inherent clinical separability of these datasets. The datasets do possess well-defined clinical features with strong predictive correlations with the target variable; these allow the application of multiple learning algorithms in order to learn to discover similar discriminative features. Additionally, the strict validation protocol adopted in the experimental design involves a two-step evaluation using independent test sets and 5-fold cross-validation which was utilized on the training data only. This ensures that the results reported are unbiased and free from data leakage. Moreover, every feature selection and oversampling process was integrated into the training folds in the process of cross-validation so that all the information contained in the validation or test sets was not used in training the models or constructing features. Besides, the relatively small variation in the performance of various model configurations can be attributed to the robustness of the preprocessing pipeline and hybrid feature-selection methods, as opposed to overfitting. The agreement between various classifiers indicates that the models are learning consistent and clinically meaningful patterns and not just memorizing noise or dataset-specific artifacts.

The research emphasizes ensemble learning as one way to achieve better predictive accuracy as well as provides empirical evidence of the robustness and stability of classification decisions. It has been demonstrated in previous works that ensemble structures are capable of significantly influencing classifier behavior during perturbations and during uncertain conditions. To illustrate, Kwon et al. (2018) [64] revealed that hierarchical ensemble structures influence the overall response of multiple classifiers, as ensemble decision mechanisms naturally precondition the model robustness, in contrast to those of individual classifiers. This finding supports the design of the proposed MSR soft-voting ensemble when heterogeneous learners MLP, SVM and RF are aggregated, suggesting that this produces more consistent and reliable predictions over multiple datasets. Within the framework of screening diabetes at an earlier stage, the ensemble robustness contributes to achieving a balance between sensitivity and specificity, thereby reducing both false negatives and false positives and improving clinical reliability. In addition to predictive performance, algorithmic efficiency is also critical in the practical application of multi-stage learning pipelines. In a related work in the literature, Kwon et al. (2024) [65] state in a prior study that evaluating the time and space complexity of different algorithmic components in a systematic way is important, instead of just using accuracy as an indicator. Based on this view, the computational cost of every step of the proposed framework, that is, preprocessing, feature selection, and ensemble learning, was analyzed. The findings indicate that despite the additional computing burdens associated with hybrid feature selection followed by ensemble integration, the total time is feasible and scalable, with considerable improvements in robustness and generalization being achieved on the Pima Indians with Diabetes, Frankfurt, and combined datasets.

The originality and efficiency of the proposed approach are highlighted through comparisons to previous studies. Conventional techniques on the Pima Indians with Diabetes dataset, including the KNN, RF, and the logistic regression, achieved accuracies ranging between 76.8 and 96.27%, often with imbalanced sensitivity and specificity. Overall, the findings highlight that achieving a dependable early detection of diabetes requires the integration of powerful preprocessing, hybrid feature selection, and optimized classifiers. Hybrid feature selection enhances feature relevance, whereas SVM demonstrates consistent performance and equal sensitivity and specificity. The proposed framework has been proven to perform at the state-of-the-art level with several datasets, which confirms its potential as a useful tool that can be used in clinical decision-making and early intervention in diabetes management.

Although recent studies increasingly focus on deep learning being used to tackle medical prediction problems, classical machine learning models are also highly applicable to structured and tabular clinical data, as is the case with this study. Practically speaking, in most healthcare applications, the models can be more interpretable, computationally efficient, and potentially more generalizable, particularly given the limited size of healthcare data files. The findings of this work show that with strong preprocessing, hybrid feature selection, and ensemble learning, traditional classifiers (i.e., SVM and RF) can achieve state-of-the-art performance in terms of sensitivity and specificity by balanced trade-off. Thus, the findings emphasize methodological rigor, robustness and clinical reliability rather than the novelties of the algorithms, which makes this approach suitable for real-world applications associated with diabetes screening. The results show that novelty in medical decision-support systems can be developed not only by using new algorithms, but also by the combination of old approaches, and by their validation and interpretation in clinically significant situations. As was shown by the proposed framework, hybrid feature selection and ensemble learning can provide strong and generalizable performance even in datasets with different statistical properties.

The combination of datasets is expected to increase the sample size and statistical power, but this may also introduce bias because the population characteristics and data collection procedures might differ. In response to this, the Pima Indians with Diabetes and Frankfurt datasets were also evaluated individually, and no significant differences in performance trends were observed. These findings are indicative of the good generalization capability of the proposed model. Nevertheless, future research will incorporate site-held-out and cross-dataset analysis to establish greater clinical strength.

Even though the performance metrics and ROC curves reveal strong classification capability, the results were obtained under strict evaluation conditions, and with unseen test data. Hence, the high performance reflects strong feature separability and sound model design rather than overfitting or data leakage. It is important to note that other feature-selection, oversampling and boosting algorithms were not considered in this study because of space and computational limitations. Further research on the topic may explore more methods and combination tactics to improve the model stability and generalization even further.

The study has limitations, such as the lack of external validation of fully independent datasets, although the model performed well in internal evaluations. Further research will focus on testing the proposed framework on external and multi-center datasets, which will further ensure the generalizability and clinical applicability of the findings.

## 5. Conclusions

In the past two decades, wearable technologies have attracted significant scientific interest in the field of healthcare, particularly for patients with chronic illnesses like diabetes. This research has been primarily focused on patients using these technologies to monitor their blood sugar levels. These approaches are able to support the management of diabetes as well as assist in the prevention of complications that are associated with the condition. In addition, the use of these devices has led to improvements, not only in the management of diabetes, but also in people’s overall quality of life. The metabolic condition known as diabetes is characterized by elevated blood-glucose levels. Improving one’s ability to diagnose diabetes at an earlier stage is the primary purpose of this investigation. Preprocessing has been investigated, and it was discovered that it plays an essential role in accurate and precise prediction. The proposed approach can be applied to a variety of feature-selection approaches and classification techniques. The MSR classifier, which is based on a voting idea, is employed to improve classification performance, and it also generates a more effective predictive model. The Pima Indians with Diabetes and Frankfurt datasets were combined and analyzed before being used to test our model. The effectiveness of the framework is demonstrated by comparing the results with state-of-the-art methods. According to the results of the performance assessment, the occurrence of diabetes could be predicted with a level of accuracy that was acceptable. The proposed model may be extended in future work to other disease prediction tasks. Additionally, the classification component of the fusion framework could be explored further by using alternative machine learning classifiers.

## Figures and Tables

**Table 1:** Previous works on diabetic prediction.

Ref	Method	Result	Drawback	Year
[21]	NB and clustering	Acc = 77.26%, Spe = 89.01%, Sen = 53.67%	The specificity dropped by 10.99%, while the sensitivity dropped by 2.33%, and there was also a small number of datasets used.	2019
[22]	SVM	Acc = 85.71%	It is recommended that the use of a dataset that contains a large number of features be given some thought in order to determine whether or not feature selection plays a vital role in enhancing accuracy during testing.	2020
[23]	KNN, logistic Gaussian, Naive Bayes, SVM-radial basis function	Acc = 83.20%	The method has not yet been validated on patient datasets of a significant scale; additionally, testing of the system ought to be conducted, along with the incorporation of additional equipment descriptions and patient information.	2021
[24]	MLP, SAE and CNN, SAE and MLP	Acc = 92.31%	The restricted number of patients in the PIDD makes it difficult to generalize these findings.	2021
[10]	RF and XG boost	Acc = 74.1%	The level of accuracy achieved by the models was significantly lower in comparison to that which was already available. In order to get the most out of the algorithm, the tuning of the hyperparameters needs to be improved.	2021
[25]	TFKNN	Acc = 90.63, Spe = 85%, Recall = 93.18%, Pre = 93.18%,	It has been determined that the limitations of the research lie in the threshold selection as well as the hyperparameter tuning. In addition, the uncertainty categorization examines each instance on its own, without taking into account any global characteristics or instances that are regarded as difficult.	2022
[26]	Generalized Boosted Regression modeling	Acc = 90.91%, Spe = 85.19%, kappa = 78.77%	It was not possible to evaluate the performance of specific classifiers using additional T2D datasets. The effectiveness of the existing system was not exhaustively compared with the performance of extended classifiers in this work.	2022
[27]	Boruta feature selection + K-Means++ + stacking ensemble	Acc = 98%	Heavy dependence on Pima (small, classic dataset); may not generalize to real clinical populations.	2023
[28]	GA-XGBoost + Stacking Ensemble, with SHAP explainability	Acc = 97.7%	Increased computational complexity; evaluated mainly on benchmark dataset.	2024
[29]	Hybrid ML models (LR, SVM, RF, ANN) for pediatric diabetes prediction	Acc = 99.98%	Limited pediatric datasets; model generalization across populations not fully validated.	2026
[30]	Optimized XGBoost with Bayesian hyperparameter optimization	Acc = 97.26% F1-score = 95.72% MCC = 81.18%	Optimization increases training time; dataset-specific tuning required.	2025
[31]	Conventional ML + Deep Learning models	Acc = 91.08%	High data and computational requirements; interpretability remains limited.	2025
[32]	Supervised ML models (LR, KNN, SVM, RF)	Acc = 96.06% Sen = 98.54% Spe = 93.63%	Moderate performance compared to ensemble/boosting approaches.	2025

**Table 2:** Description of attributes represented in the Pima Indians with Diabetes dataset and Frankfurt Diabetes dataset.

Attributes	Description	Range Values
Pregnancy	Pregnancy instances	0–17
Glucose	Test result for oral glucose tolerance in terms of milligrams	0–199
Blood pressure	Diastolic blood pressure (mm Hg)	0–122
Skin Thickness	Triceps skinfold thickness in (mm)	0–99
Insulin	Insulin in blood (mu U/mL)	0–846
BMI	Body mass index (weight in kg/(height in m)^2^)	0–67.1
Diabetes Pedigree Function	History in relatives; Genetic relation	0.078–2.42
Age	Age of the patient	21–81
Outcome	Positive or negative diabetes disease	1 or 0

**Table 3:** Hyperparameters of the models used based on the classifiers.

Model	Parameters	Value
MLP	Solver for Optimum Weight	Adam
	Activation (Hidden Layers)	ReLU
	Activation (Output Layer)	Sigmoid
	Alpha	1 × 10^−4^
	Early Stopping	True
	Number of Iterations	500
	Learning Rate Init	0.01
	Momentum	0.2
	Hidden Layers Sizes	32, 16
	Batch Size	32
SVM	Kernel	RBF
	Penalty Parameter C	1
	Class-weight	balanced
	Gamma	Scale
	Cache size	200
	Decision Function Shape	Ovr
	Tolerance	1 × 10^−3^
RF	Number of estimators	200
	Criterion	Entropy
	Bootstrap	True
	Min-samples-split	2–5
	Class-weight	balanced

**Table 4:** The distributions of the diabetic datasets used in this research.

Dataset	Number of Instances	Number of Features	Positive	Negative
Pima	768	8	268	500
Frankfurt	2000	8	684	1316
Combined	2768	8	952	1816

**Table 5:** Performance results (%) of classifiers with different preprocessing methods.

Method	Classifier	Accuracy	Sensitivity	Specificity	Precision	F1-Score
Original	MLP	77.08	77.1	71.02	76.5	76.88
	SVM	77.34	77.03	71.44	76.94	76.64
	RF	75.39	75.44	69.02	74.81	75.93
SMOTE	MLP	83.32	84.63	74.41	83.66	85.02
	SVM	76.43	75.87	73.31	75.93	75.80
	RF	90.88	90.91	72.73	90.08	91.04
ADASYN	MLP	79.29	79.96	73.54	78.95	79.51
	SVM	74.91	75.98	72.12	75.38	74.50
	RF	89.01	90.32	70.97	89.31	91.44

**Table 6:** Performance results (%) for the Pima Indians with Diabetes dataset using various models.

Feature	Classifier	Accuracy	Sensitivity	Specificity	Precision	F1-Score
GA	MLP	80.68	81.32	79.54	82.85	81.25
	SVM	82.51	83.92	81.72	84.73	83.49
	RF	85.25	86.12	84.84	87.78	86.17
	MSR	88.72	89.54	88.55	87.18	86.79
CFS	MLP	78.23	79.81	77.13	80.25	78.16
	SVM	79.91	81.03	78.71	81.32	80.11
	RF	82.76	83.61	82.93	83.47	83.34
	MSR	85.41	86.93	84.19	87.73	88.73
RFE	MLP	81.89	83.98	82.18	84.92	83.22
	SVM	84.87	85.99	84.52	85.68	84.16
	RF	87.22	86.36	86.54	89.22	88.92
	MSR	89.91	90.11	89.22	91.37	92.87

**Table 7:** Performance results (%) for the Frankfurt dataset using various models.

Feature	Classifier	Accuracy	Sensitivity	Specificity	Precision	F1-Score
GA	MLP	79.85	80.92	78.74	81.32	80.12
	SVM	81.42	82.75	80.11	83.12	82.05
	RF	84.03	85.12	83.14	86.18	85.65
	MSR	87.15	88.32	86.74	87.55	87.93
CFS	MLP	77.92	78.61	77.11	79.02	78.31
	SVM	79.16	80.21	78.07	80.38	79.29
	RF	82.24	83.14	81.32	83.67	83.4
	MSR	85.01	86.15	84.32	86.42	85.83
RFE	MLP	81.03	82.12	80.21	83.05	82.58
	SVM	83.22	84.18	82.47	84.57	84.37
	RF	86.14	86.92	85.37	88.05	87.45
	MSR	89.38	90.21	88.74	91.12	90.67

**Table 8:** Performance results (%) for the combined dataset, using various models.

**Feature**	**Classifier**	**Accuracy**	**Sensitivity**	**Specificity**	**Precision**	**F1-Score**
GA	MLP	80.68	81.32	79.54	82.85	81.25
	SVM	82.51	83.92	81.72	84.73	83.49
	RF	85.25	86.12	84.84	87.78	86.17
	MSR	88.72	89.54	88.55	87.18	86.79
CFS	MLP	78.23	79.81	77.13	80.25	78.16
	SVM	79.91	81.03	78.71	81.32	80.11
	RF	82.76	83.61	82.93	83.47	83.34
	MSR	85.41	86.93	84.19	87.73	88.73
RFE	MLP	81.89	83.98	82.18	84.92	83.22
	SVM	84.87	85.99	84.52	85.68	84.16
	RF	87.22	86.36	86.54	89.22	88.92
	MSR	89.91	90.11	89.22	91.37	92.87

**Table 9:** Average classification performance results (%) before and after the application of various feature-selection methods.

Feature-Selection Method	No. of Features	Accuracy	Sensitivity	Specificity	Precision	F1-Score
Without feature section	8	85.77	83.81	84.16	84.12	83
CFS	6	87.78	85.67	86.42	86.52	85.2
RFE	7	89.29	88.69	88.09	88.71	88.72
GA	6	91.04	91.42	90.72	90.24	91.37

**Table 10:** Evaluation results (%) for feature selection: GA + RFE.

Classifier	Accuracy	Sensitivity	Specificity	Precision	F1-Score
MLP	96.4	94.73	97.54	97.05	96.31
SVM	98.2	97.03	99.37	99.07	98.26
RF	95.8	95.21	96.22	96.36	95.15
MSR	97.1	96.43	98.11	98.14	97.81

**Table 11:** Evaluation results (%) for feature selection: GA + CFS.

Classifier	Accuracy	Sensitivity	Specificity	Precision	F1-Score
MLP	95.5	93.23	96.91	96.08	94.25
SVM	97.6	96.71	98.62	98.71	97.07
RF	94.9	94.66	95.73	95.77	94.05
MSR	96.5	95.13	97.06	97.19	96.33

**Table 12:** Evaluation results (%) for feature selection: REF + CFS.

Classifier	Accuracy	Sensitivity	Specificity	Precision	F1-Score
MLP	95.9	94.41	96	96.82	95.41
SVM	98.0	97.43	99.03	99.51	98.72
RF	95.3	94.07	95.05	96.39	94.17
MSR	97.0	96.12	97.18	98.42	97.06

**Table 13:** G-Mean values of classifiers with combined feature-selection methods.

Classifier	GA + RFE	GA + CFS	RFE + CFS
MLP	0.96	0.95	0.94
SVM	0.98	0.96	0.96
RF	0.955	0.945	0.947
MSR	0.98	0.97	0.97

**Table 14:** Ablation showing contributions of feature selection and MSR ensemble.

Component	Accuracy	Sensitivity	Specificity	Precision	F1-Score
Base SVM	86.0	83.06	87.1	86.72	85.16
Imputation	87.2	85.21	87.8	88.0	86.51
Oversampling	88.0	86.5	88.23	89.02	87.7
Standardization	88.42	87.43	89.29	88.01	88.3
GA	90.02	89.23	90.2	91.02	91.71
CFS	90.14	89.47	91.22	91.05	90.91
RFE	91.65	90.77	92.09	92.43	91.88
GA + CFS	94.37	93.77	94.99	94.87	94.19
GA + RFE	95.54	94.81	95.29	96.01	95.17
RFE + CFS	98.0	97.14	98.09	98.33	98.37
Ensemble + MSR	98.3	97.6	99.1	99.6	98.8

**Table 15:** Comparative analysis among the proposed methods and previous studies.

Study	Method	Dataset	Accuracy	Sensitivity	Specificity	Precision	F1-Score	Year of Publication
[55]	KNN	Pima	88	90	N/A	87	88	2019
[57]	RF	Pima	87	85	N/A	81	83	2019
[60]	SMO	Pima	77.34	77.30	N/A	76.90	76.30	2020
[14]	SVM	Pima	83.2	N/A	N/A	N/A	76	2021
[15]	DT + SMOTE + GA	Pima	82.12	N/A	N/A	N/A	N/A	2021
[53]	LR	Pima	76.8	76.8		73.3		2021
[56]	KNN	Pima	92.28	N/A	N/A	N/A	N/A	2021
[13]	NN	Pima	88.60	N/A	N/A	N/A	N/A	2021
[25]	TFKNN	Pima	90.63	93.18		93.18	93.18	2022
[1]	DL	Pima	95	95		90	93	2022
[3]	RF	NHANES	94.25	N/A	N/A	N/A	N/A	2019
[46]	SVM + ANN	Combined	94.67	89.23	97.32	94.19	N/A	2021
[54]	DCSGAN	Pima	96.27	96.98		96.98	96.98	2023
[58]	Multi-Layer NN	Pima	97	97	95	N/A	N/A	2023
[61]	SHAP	Pima	94.67	N/A	N/A	N/A	95.95	2023
[59]	Ensemble	Pima + Frankfurt	98.79	N/A	N/A	N/A	N/A	2024
[62]	XGBoost	Clinical Diabetes Dataset	86.87	84.4	89.12	88.9	86.44	2025
	LightGBM	Clinical Diabetes Dataset	86.78	84.37	88.88	88.72	86.35	2025
[27]	DNN	Pima	98.16	N/A	N/A	N/A	N/A	2023
[63]	Ensemble Learning	Pima	99	100	N/A	98	99	2025
Proposed	All feature + MSR	Pima	89.91	90.11	89.22	91.37	92.87	Ours
	All feature + MSR	Frankfurt	89.38	90.21	88.74	91.12	90.67	
	All feature + MSR	Combined	89.91	90.11	89.22	91.37	92.87	
	GA + RFE + SVM	Combined	98.2	97.03	99.37	99.07	98.26	
	GA + CFS + SVM	Combined	97.6	96.71	98.62	98.71	97.07	
	REF + CFS + SVM	Combined	98.0	97.43	99.03	99.51	98.72	

## Data Availability

The data supporting the findings of this study are available via the link provided in Section 3.1.
